# In Vivo ETosis of Human Eosinophils: The Ultrastructural Signature Captured by TEM in Eosinophilic Diseases

**DOI:** 10.3389/fimmu.2022.938691

**Published:** 2022-07-07

**Authors:** Vitor H. Neves, Cinthia Palazzi, Kennedy Bonjour, Shigeharu Ueki, Peter F. Weller, Rossana C. N. Melo

**Affiliations:** ^1^ Laboratory of Cellular Biology, Department of Biology, Institute of Biological Sciences, Federal University of Juiz de Fora, Juiz de Fora, Brazil; ^2^ Laboratory of Molecular and Morphological Pathology, Department of Pathology, School of Veterinary Medicine and Animal Science, University of São Paulo, São Paulo, Brazil; ^3^ Department of General Internal Medicine and Clinical Laboratory Medicine, Graduate School of Medicine, Akita University, Akita, Japan; ^4^ Department of Medicine, Beth Israel Deaconess Medical Center, Harvard Medical School, Boston, MA, United States

**Keywords:** eosinophil, eosinophil-associated diseases, eosinophil extracellular traps, eosinophilic chronic rhinosinusitis, ulcerative colitis, hypereosinophilic syndrome, transmission electron microscopy, Charcot-Leyden crystals

## Abstract

Eosinophilic diseases, also termed eosinophil-associated diseases (EADs), are characterized by eosinophil-rich inflammatory infiltrates and extensive eosinophil degranulation with clinically relevant organ pathology. Recent evidence shows that eosinophil cytolytic degranulation, that is, the release of intact, membrane-delimited granules that arises from the eosinophil cytolysis, occurs mainly through ETosis, meaning death with a cytolytic profile and extrusion of nucleus-originated DNA extracellular traps (ETs). The ultrastructural features of eosinophil ETosis (EETosis) have been studied mostly *in vitro* after stimulation, but are still poorly understood *in vivo*. Here, we investigated in detail, by transmission electron microscopy (TEM), the ultrastructure of EETosis in selected human EADs affecting several tissues and organ systems. Biopsies of patients diagnosed with eosinophilic chronic rhinosinusitis/ECRS (frontal sinus), ulcerative colitis/UC (intestine), and hypereosinophilic syndrome/HES (skin) were processed for conventional TEM. First, we found that a large proportion of tissue-infiltrated eosinophils in all diseases (~45-65% of all eosinophils) were undergoing cytolysis with release of free extracellular granules (FEGs). Second, we compared the morphology of tissue inflammatory eosinophils with that shown by *in vitro* ETosis-stimulated eosinophils. By applying single-cell imaging analysis, we sought typical early and late EETosis events: chromatin decondensation; nuclear delobulation and rounding; expanded nuclear area; nuclear envelope alterations and disruption; and extracellular decondensed chromatin spread as ETs. We detected that 53% (ECRS), 37% (UC), and 82% (HES) of all tissue cytolytic eosinophils had ultrastructural features of ETosis in different degrees. Eosinophils in early ETosis significantly increased their nuclear area compared to non-cytolytic eosinophils due to excessive chromatin decondensation and expansion observed before nuclear envelope disruption. ETosis led not only to the deposition of intact granules, but also to the release of eosinophil sombrero vesicles (EoSVs) and Charcot-Leyden crystals (CLCs). Free intact EoSVs and CLCs were associated with FEGs and extracellular DNA nets. Interestingly, not all cytolytic eosinophils in the same microenvironment exhibited ultrastructure of ETosis, thus indicating that different populations of eosinophils might be selectively activated into this pathway. Altogether, our findings captured an ultrastructural signature of EETosis *in vivo* in prototypic EADs highlighting the importance of this event as a form of eosinophil degranulation and release of inflammatory markers (EoSVs and CLCs).

## Introduction

Eosinophil-associated diseases (EADs), also termed eosinophilic diseases, encompass a broad spectrum of diseases of different etiologies characterized by the presence of eosinophil-rich inflammatory infiltrates and/or widespread extracellular deposition of eosinophil-derived products. EADs include allergies, gastrointestinal disorders, dermatoses, hyperosinophilic syndromes (HESs), infectious diseases, and neoplastic disorders and can affect multiple organs [reviewed in (1, 2)].

A common feature of EADs is the extensive eosinophil degranulation in different target organs resulting in clinically relevant organ pathology. Despite the progress in the field, the precise roles of eosinophils in the pathogenesis of EADs are not well understood (3). For decades, we have been applying transmission electron microscopy (TEM) to understand the degranulation/secretory activities of eosinophils in human biopsy specimens from patients with EADs. TEM can unequivocally identify eosinophils and their tissue-deposited granules, referred to as free extracellular granules (FEGs), without the use of a specific marker because eosinophils have a unique ultrastructure [reviewed in (4–6)]. Moreover, TEM is also a premier technique to distinguish among the different modes of eosinophil degranulation, which can be clearly visualized only at high microscopic resolution (6).

More recently, our group identified that human eosinophils degranulate through ETosis, introducing the term eosinophil ETosis (EETosis) to describe a cytolytic mode of eosinophil death with extrusion of nucleus-originated DNA extracellular traps (ETs) similar to NETosis (7). By using different stimuli such as calcium ionophore, phorbol myristate acetate (PMA), immobilized immunoglobulins, and platelet-activating factor (PAF) in combination with cytokines, this work demonstrated molecular and morphological eosinophil features similar to those described for neutrophils undergoing this process of death (7). EETosis has now been increasingly recognized in tissues and secretions from patients with diseases such as eosinophilic chronic rhinosinusitis (ECRS) (8), hypereosinophilic syndrome (HES) (9), eosinophilic granulomatosis with polyangiitis (EGPA) (10), eosinophilic otitis media (EOM) (11), and chronic obstructive pulmonary disease (COPD) (12).

The ultrastructural features of EETosis have been mainly demonstrated *in vitro* after cell stimulation, but they are still poorly understood *in vivo*. Indeed, a limited number of studies have been applying TEM to study EETosis in organs from patients with eosinophilic diseases, specifically in EGPA (10) and HES (7, 9), which provided initial evidence supporting this type of cytolytic cell death *in vivo*.

Here, we investigated by transmission electron microscopy (TEM) the ultrastructural characteristics of EETosis in selected human EADs [ECRS, ulcerative colitis (UC), and HES] affecting several tissues (frontal sinus, intestine, and skin, respectively) and organ systems. By performing a comprehensive qualitative and quantitative TEM study of human biopsies, we captured the ultrastructural signatures of EETosis, confirming and highlighting this kind of cell death as an important event associated with eosinophil degranulation during inflammatory diseases.

## Material and Methods

### Ethics Statement

This study was carried out in accordance with the ethical principles taken from the Declaration of Helsinki and written informed consent was obtained from donors. Institutional Review Board (IRB) approval was obtained annually from the Beth Israel Deaconess Medical Center Committee on Clinical Investigation (Boston, MA, USA), with the most recent IRB-approved protocol number 2001P000561.

### Biopsy Samples

Tissue biopsy samples were obtained from patients diagnosed with typical eosinophilic diseases during clinical evaluations. The specimens were obtained from: i) the nasal sinuses of two patients with ECRS; ii) the ileum, rectum or continent pouches of 16 patients with UC with traditional surgical pathology criteria of the resected specimens used for diagnosis; and iii) skin lesions of two patients with HES negative for FIP1-like 1/platelet-derived growth factor-alpha mutation.

### Eosinophil Isolation

Eosinophils were purified from the peripheral blood from healthy donors using negative selection, as before (13). Briefly, venous blood was collected in a 6% dextran saline solution, and RBCs were allowed to sediment. Buffy coat was centrifuged over Ficoll-Paque (GE Healthcare, Pittsburgh, Pa) to separate granulocyte pellets. Eosinophils were isolated by means of incubation with a depletion antibody cocktail (StemSep; STEMCELL Technologies, Vancouver, British Columbia, Canada), followed by passage over magnetized columns (Miltenyi Biotec, Auburn, Calif). The purity of isolated eosinophils was greater than 98% of nucleated cells, and viability was greater than 99%.

### Induction of *In Vitro* EETosis

Purified human eosinophils (1×10^6^/mL) were seeded in eight-well LAB-TEK II chamber slides (Nunc, Roskilde, Denmark) and stimulated with PAF (1 μM; Enzo Life Sciences, Farmingdale, NY) and recombinant human interleukin-5 (IL-5) (10 ng/mL; catalog number 205-IL, R&D Systems, Minneapolis, MN), or IL-5 alone in phenol-red free RPMI 1640 medium containing 0.3% bovine serum albumin (BSA; Sigma) at 37°C for 180 min (7, 14).

### Immunofluorescence

Detections of released EETs were done per previous works from our group (7, 14). Stimulated eosinophils as above were fixed with 4% paraformaldehyde for 10 min at room temperature (RT). The slides were subsequently incubated with primary rabbit anti-citrullinated H3 histone (CitH3) monoclonal antibody (10 μg/mL, 90 min at room temperature; Abcam). Alexa-488-conjugated secondary antibodies (Life Technologies) were then added for 30 min at RT. Isotype-matched control antibodies and Hoechst 33342 were used in each experiment. Samples were mounted using Prolong Diamond (Life Technologies) and images were obtained using a LSM 780 confocal microscope (Carl Zeiss, Oberkochen, Germany).

### Transmission Electron Microscopy

Samples for TEM (eosinophils isolated from the peripheral blood and tissue fragments) were prepared per protocols established by our group (15). Blood eosinophils, stimulated or not, were fixed in a mixture of freshly prepared aldehydes [final concentration of 1% paraformaldehyde and 2.5% glutaraldehyde (EM grade, 50% aqueous, Electron Microscopy Sciences-EMS, Hatfield, PA)] in 0.1 M sodium cacodylate buffer, pH 7.4 for 1h at RT. Biopsy samples (skin, intestines, and nasal sinuses) were fixed for 4h at RT using the same fixative. After washing with sodium maleate buffer, pH 5.2, samples were stained en bloc in 2% uranyl acetate in 0.05 M sodium maleate buffer, pH 6.0 for 2 h at RT and washed in the same buffer as before prior to dehydration in graded ethanols and infiltration and embedding with a propylene oxide-Epon sequence (Eponate 12 Resin; Ted Pella, Redding, CA). Alternatively, additional samples were post- fixed in 2% aqueous osmium tetroxide and 1.5% potassium ferrocyanide in 0.1 M sodium phosphate buffer, pH 6.0 (reduced osmium) before dehydration and embedding as above. After polymerization at 60°C for 16 h, thin sections were cut using a diamond knife on an ultramicrotome (Leica, Bannockburn, IL). Sections were mounted on uncoated 200-mesh copper grids (Ted Pella) before staining with lead citrate and viewed with a transmission electron microscope (CM 10; Philips, or Tecnai G2-20-ThermoFischer Scientific/FEI 2006, Eindhoven, the Netherlands) at 80-120 KV.

### Quantitative TEM Analyses

To investigate tissue eosinophils and their processes of secretion in different organs, electron micrographs showing infiltrated eosinophils were randomly acquired (total n = 397 electron micrographs) and a total of 66,000 μm² of tissue were analyzed (17,000 μm² for nasal sinuses, 29,000 μm^2^ for intestines, and 20,000 μm^2^ for skin) in biopsies of patients with ECRS, UC, and HES respectively. All eosinophils found in the tissue areas, including regions with FEGs, were scored. Secretory processes were identified and quantitated as piecemeal degranulation (PMD); classical/compound exocytosis, or cytolysis as described (16); and the percentages of these processes were enumerated.

To evaluate early morphological signs of ETosis the following aspects were quantitated: i) Chromatin expansion: the nuclear areas (μm^2^) were measured within eosinophils (total n= 265 cells) and the areas of nuclei with clear euchromatin/heterochromatin distinction (n=212 cells) were compared with those showing only euchromatin (n= 53 cells); ii) Diameters of vesicles originated from the nuclear envelope in eosinophils with early signs of ETosis; iii) Frequency of cytolytic eosinophils with early signs of ETosis (nuclei with decondensation, delobulation, and rounding), late signs (disrupted plasma membrane and extruded decondensed chromatin), or non-ETosis (nuclei with euchromatin/heterochromatin) in each disease; iv) Numbers of free released EoSVs in inflammatory sites with late signs of EETosis; v) Proportions of free EoSVs in contact with or close to (distance <1µm) extruded decondensed chromatin as well as free intact EoSVs spread in the inflammatory tissue site (localized in areas >1µm of distance from released DNA strings); vi) long axis diameter and area of Charcot-Leyden Crystals (CLCs). All quantitative analyses were performed using Fiji software (National Institutes of Health, Bethesta, MD, USA).

### Statistical Analyses

The results are presented as mean ± SEM and significance was considered as *P* < 0.05. All data (except the comparison of CLCs’areas among groups) were analyzed with the Student’s t test, using the software GraphPad Prism version 7.00 (GraphPad Software, La Jolla California, www.graphpad.com). For the analyses focused on the areas of CLCs, we compared the groups by using Kruskal Wallis test followed by Dunn’s test to adjust for multiple comparisons.

## Results

### Eosinophils Degranulate Through PMD and Cytolysis in Prototypic EADs

First, we performed a comprehensive ultrastructural analysis to capture infiltrating eosinophils in the inflamed tissues and to understand how these cells degranulate *in vivo* in representative human EADs (ECRS, UC, and HES) affecting varied tissues (nasal sinus, intestine, and skin, respectively). By analyzing vast tissue areas (total of 66,000 μm^2^) in random thin sections, we scored all tissue eosinophils based on their typical ultrastructural features of PMD, cytolysis, or exocytosis as described [reviewed in (6, 16, 17)].

**Figure 1:**
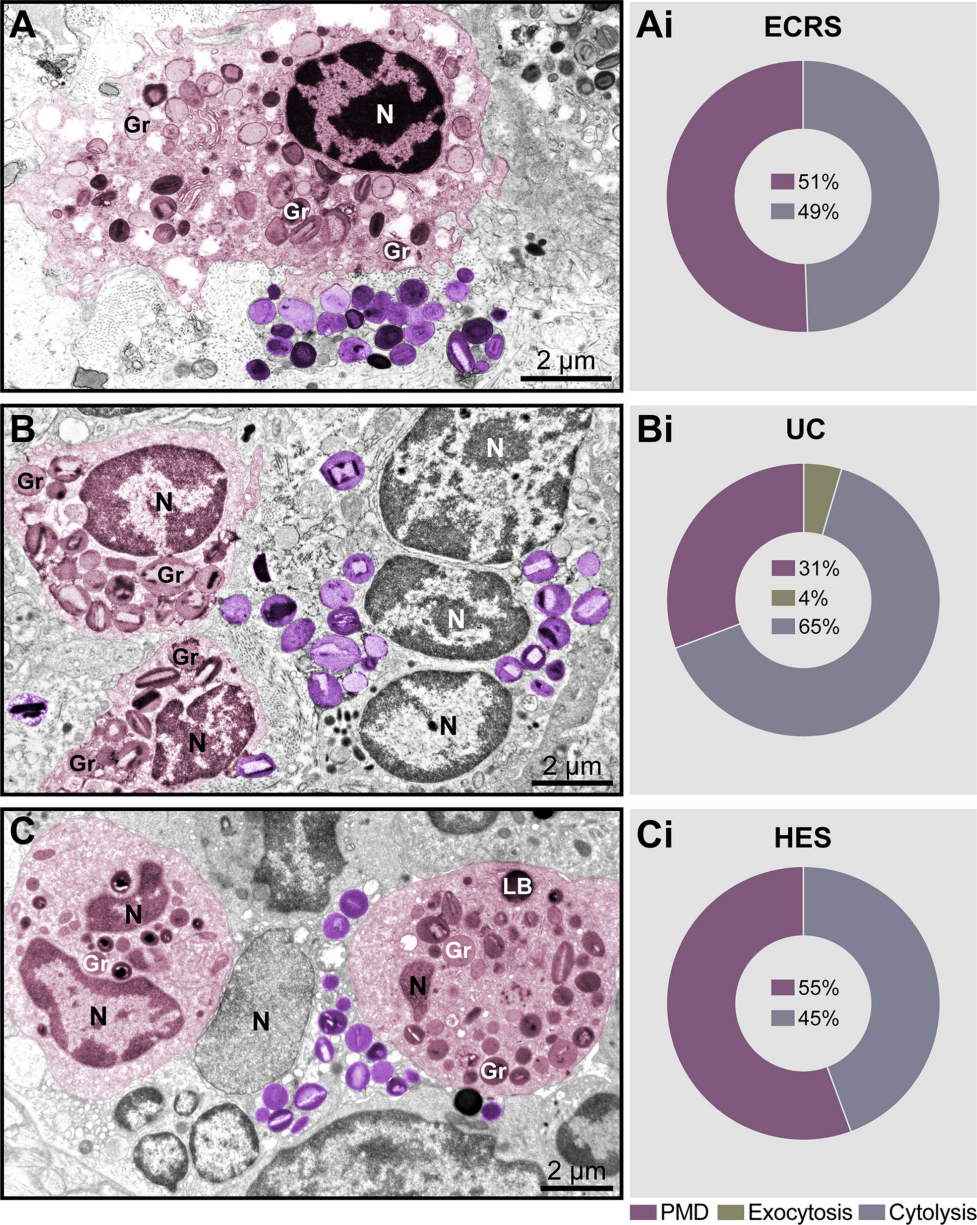
Piecemeal degranulation (PMD) and cytolysis are the main modes of eosinophil secretion in human eosinophil-associated diseases (EADs). **(A–C)** Representative micrographs of tissue eosinophils in the lamina propria of nasal sinuses (eosinophilic chronic rhinosinusitis/ECRS), lamina propria and submucosa of intestines (ulcerative colitis/UC), and papillary dermis (hypereosinophilic syndrome/HES), respectively. Eosinophils undergoing (PMD) (colored in pink) and cytolysis with deposition of free extracellular granules (FEGs, purple) are observed in the same field. Biopsy samples were prepared for TEM as before (Melo et al., 2005). **(Ai–Ci)** Quantitative analyses demonstrate predominance of PMD and cytolysis in representative EADs. A total of 335 eosinophils (99 for ECRS, 156 for UC and 80 for HES) were randomly evaluated in a total of 66,000 μm² of tissue area from two patients with ECRS (n = 2), UC (n = 16), and HES (n = 2). Gr, secretory granules; N, nucleus.

Significant tissue eosinophilia with the presence of both intact and cytolytic (disrupted) eosinophils, isolated or in clusters, was observed in the lamina propria of nasal sinuses (ECRS), lamina propria and submucosa of the intestine (UC), and dermis (HES) (**Figures 1A–C**, see also **Supplementary Figure S1**). Quantitative analyses showed that PMD and cytolysis with the release of FEGs were the predominant secretory processes observed in inflammatory eosinophils participating in all diseases (**Figures 1Ai–Ci**) while exocytosis (**Supplementary Figure S2**) was also detected in a small proportion just in the intestinal biopsies of patients with UC (**Figure 1Bi**). Resting eosinophils, which are characterized by the predominance of intact, non-mobilized specific granules (with no evidence of granule content losses) were not observed.

Having found a large proportion of eosinophils undergoing different degrees of cytolysis in all EADs (49%, 65%, and 45% in ECRS, UC, and HES, respectively, n= 335 cells) (**Figures 1Ai–Ci**), and considering that EETosis is a form of lytic death, we next evaluated eosinophils in great detail to detect morphological characteristics of EETosis. We questioned if tissue inflammatory eosinophils had the same ultrastructural hallmarks of ETosis as exhibited by eosinophils *in vitro* after stimulation with ETosis inducers.

### *In Vitro* Ultrastructural Halmarks of EETosis

**Figure 2:**
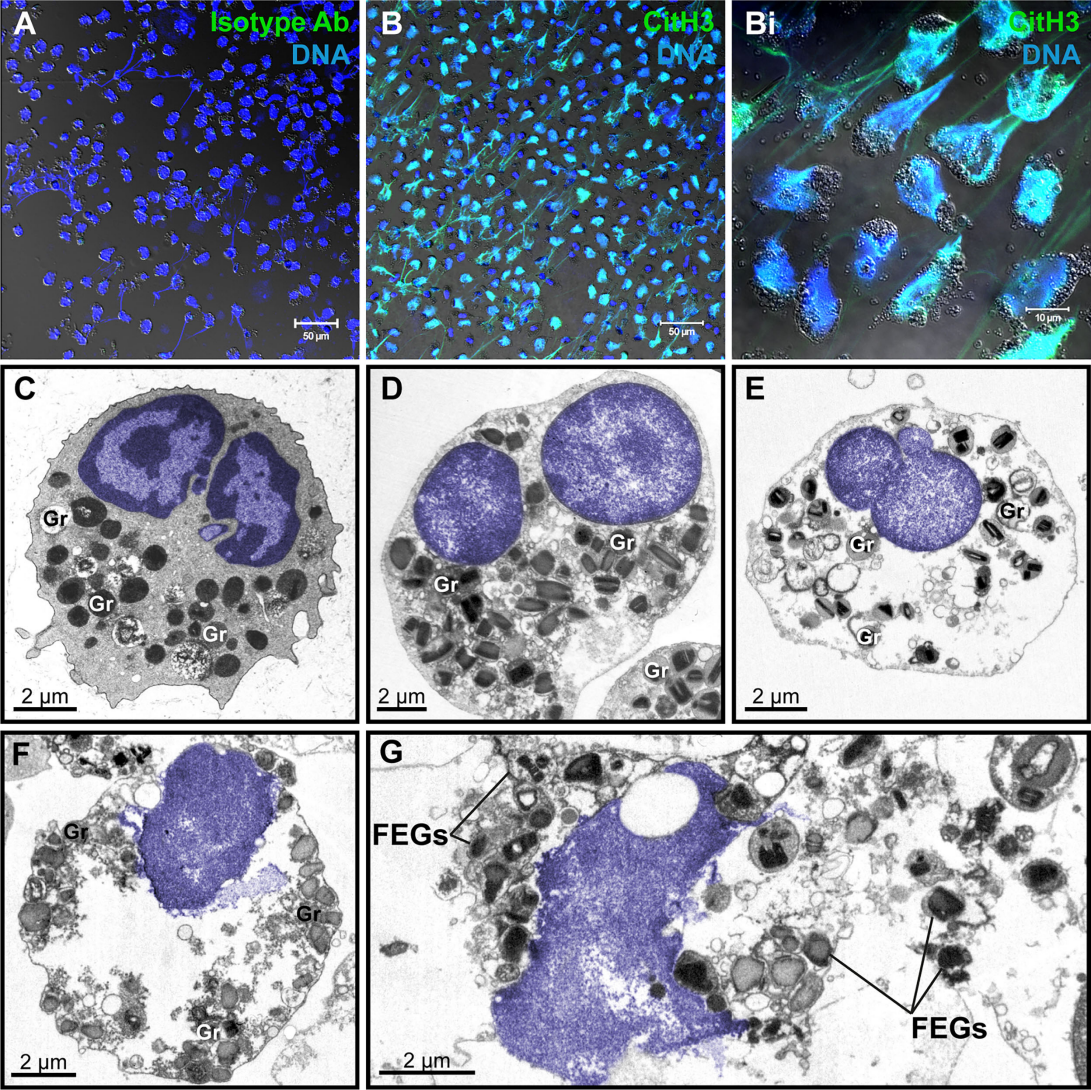
Human eosinophil ETosis observed *in vitro* after stimulation. **(A, B)** Immunofluorescence staining for isotype **(A)** or anti-citrullinated H3 histone (CitH3) antibodies(green). EETs and CitH3 are colocalized as seen by confocal fluorescence microscopy after staining for DNA (Hoechst, blue) and CitH3 (green). **(Bi)** Merged images of CitH3 + DNA-stained eosinophils in higher magnification. Differential interference contrast (DIC) images were obtained by confocal microscopy. Purified eosinophils were stimulated with PAF + IL-5 followed by fixation after 180 min. **(C)** Representative micrograph of an intact control eosinophil, stimulated with IL-5 alone, displaying a typical bilobed nucleus (colored in blue) with clear distinction between euchromatin and heterochromatin. **(D–G)** Ultrastructural alterations displayed by human eosinophils in process of ETosis after stimulation. Decondensation, delobulation, and rounding are observed. In **(F, G)**, the nuclear envelope is partially disrupted and chromatin is being released as extracellular traps. Free extracellular granules (FEGs) are deposited in the extracellular medium. Samples were prepared for TEM. Nucleus and extracellular chromatin are colored in blue. Ab, antibody; Gr, secretory granules.

To study the ultrastructure of EETosis *in vitro*, human eosinophils isolated from the peripheral blood were stimulated with a combination of eosinophil agonists (IL-5 + PAF), an efficient trigger of EETosis (7, 14). First, the release of ETs was confirmed by immunofluorescence using an antibody against DNA-citrullinated histone (CitH3), which consistently labeled DNA strings in the extracellular medium in parallel with a negative labeling using an isotype antibody control (**Figures 2A, B, 2Bi**), as previously demonstrated by our group (7, 14). Second, stimulated eosinophils were processed for TEM using a protocol for optimal morphologic preservation.

As expected, at the ultrastructural level, *in vitro* EETosis was characterized by dramatic nuclear changes. The nucleus of human mature eosinophils shows typically a bilobed appearance and a clear distinction between heterochromatin (condensed chromatin, mostly located marginally) and euchromatin (electron-lucent chromatin, more centrally localized) (**Figure 2C**). Early signs of ETosis, as observed *in vitro*, include loss of such distinction, which results in a nucleus homogeneously composed of euchromatin in parallel to a process of delobulation and rounding, in which the cell loses their original shape (**Figures 2D, E**). Chromatin expansion (**Figure 2F**), with subsequent rupture of the nuclear envelope and plasma membrane then allows the release of filamentous euchromatin structures (ETs) and FEGs to the surrounding microenvironment (**Figure 2G**). Interestingly, FEGs released during *in vitro* EETosis retain their morphology and contents or show little evidence of loss of their protein compounds (**Figure 2G**).

### Early Signs of EETosis Captured *In Vivo* by TEM

**Figure 3:**
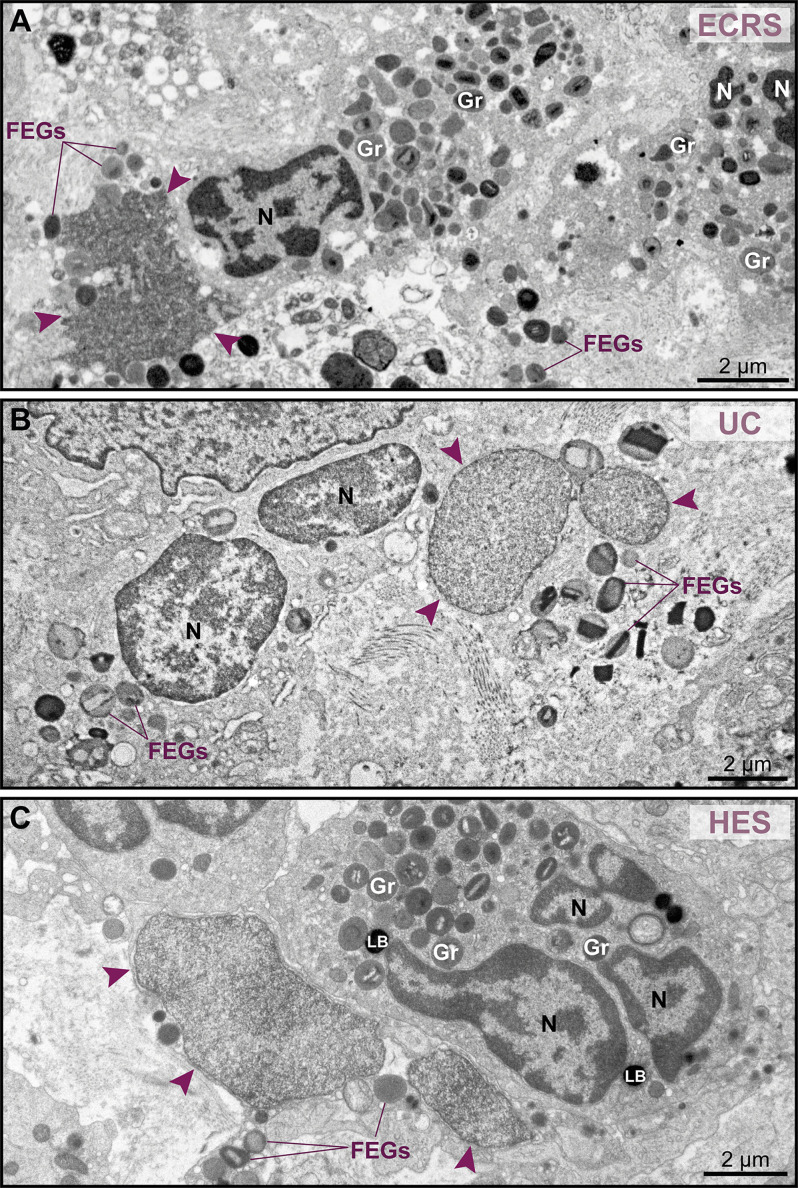
Tissue eosinophils undergoing ETosis are seen in inflammatory sites in the proximity of intact eosinophils. **(A–C)** Electron micrographs of biopsy samples from eosinophilic chronic rhinosinusitis (ECRS), ulcerative colitis (UC) and hypereosinophilic syndrome (HES) patients, respectively, show eosinophils with nuclear changes typical of ETosis, characterized by chromatin decondensation, delobulation, rounding and expansion. Note that, while the nuclei (N) of intact eosinophils show typical distinction between euchromatin/heterochromatin, the nuclei (arrowheads) of ETotic eosinophils show only euchromatin. In **(A)**, the nuclear envelope was disrupted. Samples were collected from two patients with ECRS (n = 2), UC (n = 16), and HES (n = 2) and prepared for TEM. Gr, secretory granules; FEGs, free extracellular granules. LB, lipid body.

**Figure 4:**
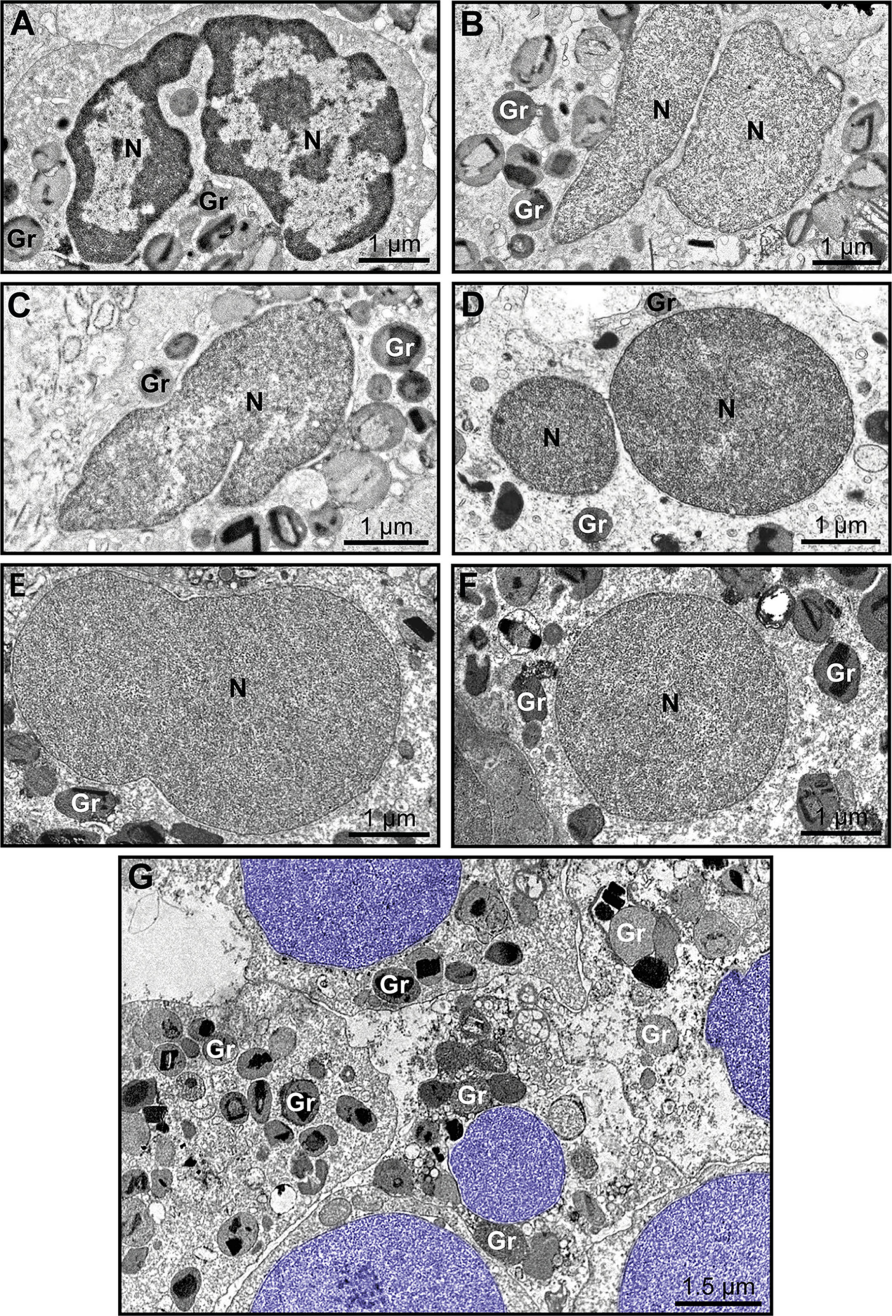
Early ultrastructural signs of ETosis in tissue inflammatory eosinophils. **(A)** A representative intact eosinophil displaying typical bilobed nucleus (N) with clear distinction between euchromatin (electron-lucent) and heterochromatin (electron-dense). **(B–F)** Decondensed nuclei (N), with homogenously distributed euchromatin, and in different stages of delobulation and rounding denote, early ETosis. While in **(B, C)** nuclear segmentation is still partially observed, **(D–F)** show nuclei in advanced process of delobulation and rounding. In **(G)**, a tissue area with several decondensed, completely round nuclei (colored in purple). Biopsy intestinal samples from patients with ulcerative colitis (n= 16) prepared for TEM. Gr, secretory granules.

Because nuclear decondensation, delobulation, and rounding (DDR) are considered canonical initial features of ETotic death preceding chromatin externalization as ETs, as described *in vitro* in both neutrophils (18) and eosinophils (19, 20), we sought these events in tissue inflammatory eosinophils. Eosinophils exhibiting DDR were detected in tissue sites from all EADs in conjunction with eosinophils showing normal nuclear ultrastructure (**Figure 3**, see also **Supplementary Figure S3**). Considering that DDR are dynamic events in ETotic eosinophils, by analyzing vast tissue areas, we found eosinophil nuclei at different stages of delobulation and rounding (**Figure 4**). Decondensation is likely preceding delobulation events since all nuclei that have lost their shape exhibited homogenously distributed euchromatin (**Figure 4**, see also **Supplementary Figure S3**).

**Figure 5:**
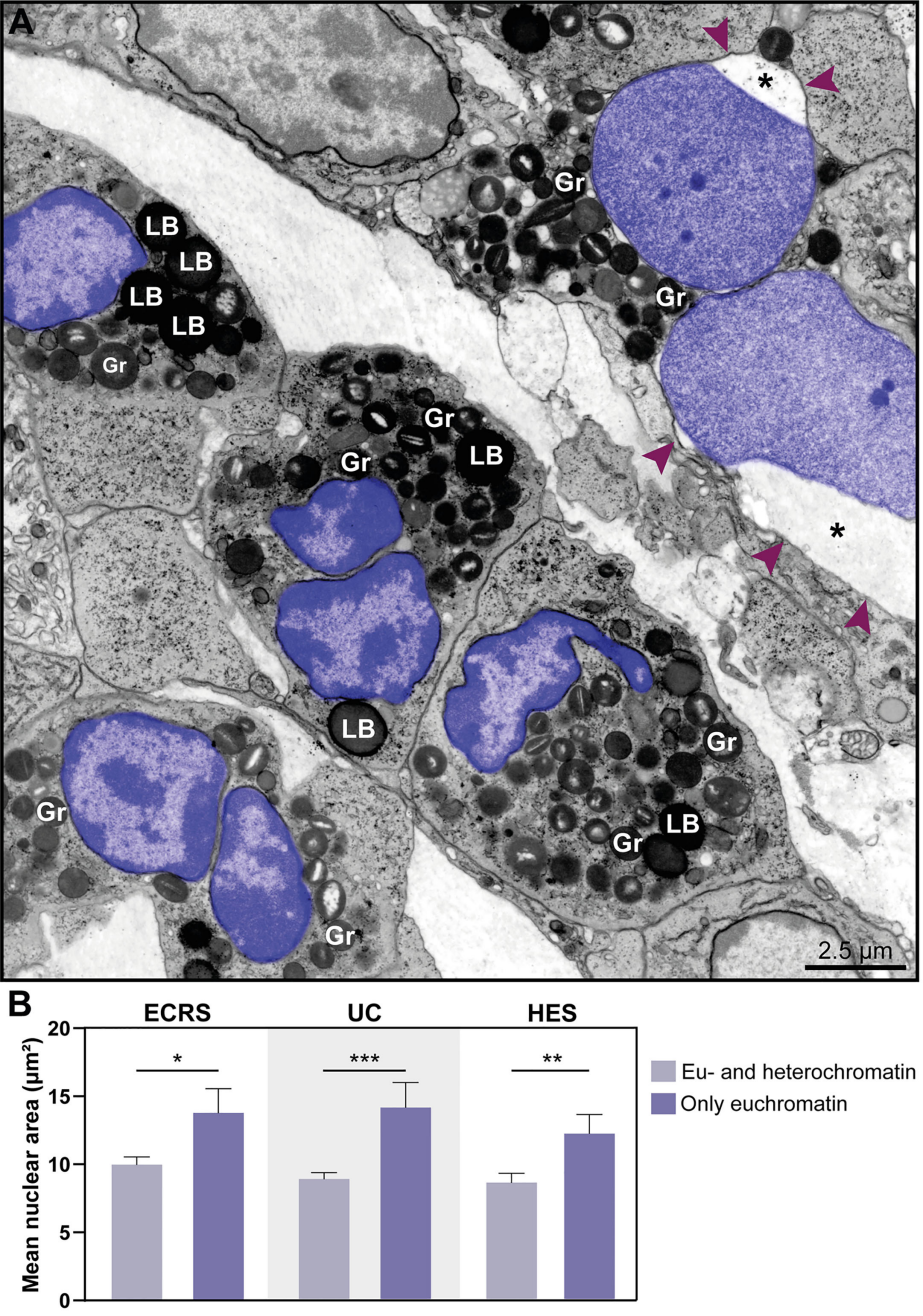
The nuclear area increases in eosinophil undergoing ETosis. **(A)** A cluster of tissue eosinophils infiltrated in the skin of a patient with hypereosinophilic syndrome (HES). While several intact eosinophils show nuclei with normal chromatin appearance (marginal heterochromatin and more internal euchromatin), an early ETotic eosinophil (upper right side) shows enlarged decondensed nucleus undergoing delobulation and rounding. Note that the nuclear space (*) of the nuclear envelope is dilated with separation of the external membrane (arrowheads) from the internal one. All eosinophil nuclei were colored in purple. In **(B)**, quantitative analyses reveal significantly higher nuclear area in eosinophils in early ETosis (only euchromatin) compared to eosinophils with euchromatin/heterochromatin distinction. A total of 263 eosinophils [77 for eosinophilic chronic rhinosinusitis (ECRS), 120 for ulcerative colitis (UC), and 68 for HES] were evaluated and the nuclear area (µm²) measured. Samples were collected from patients with ECRS (n=2), UC (n=16), and HES (n=2) and prepared for TEM. Results are expressed as means ± SEM (*P < 0.05; ***P < 0.01; ***P < 0.001). Gr, secretory granules; LB, lipid bodies.

Another distinctive nuclear alteration associated with both NETosis (21) and EETosis (19, 20) and reported *in vitro* is the chromatin expansion/swelling while within the cytoplasm and with the nuclear envelope mostly preserved. We questioned if such alteration could be detected *in vivo*. Indeed, we captured chromatin expansion in tissue eosinophils and this phenomenon drew our attention especially when eosinophils with these early signs of ETosis were close to eosinophils with normal nuclear ultrastructure (**Figure 5A**). To better document this event, we measured and compared the nuclear area between cells with normal (clear euchromatin/heterochromatin distinction) and totally decondensed (only euchromatin) nuclei for each disease. By evaluating a total of 265 eosinophils, we found that the nuclear area (μm^2^) was significantly higher in eosinophils with nuclei composed of just euchromatin than in eosinophils with normal nuclei (**Figure 5B**). Means ± SEM are: i) ECRS: 10.12 ± 0.59 (normal, n=69) versus 13.93 ± 1.80 (decondensed, n=8); *P*< 0.05;ii) UC: 8.97 ± 0.47 (normal, n=95) versus 14.23 ± 1.84 (decondensed, n=25); *P*< 0.001; iii) HES: 8.71 ± 0.69 (normal, n=48) versus 12.24 ± 1.19 (decondensed, n=20); *P*< 0.01.

**Figure 6:**
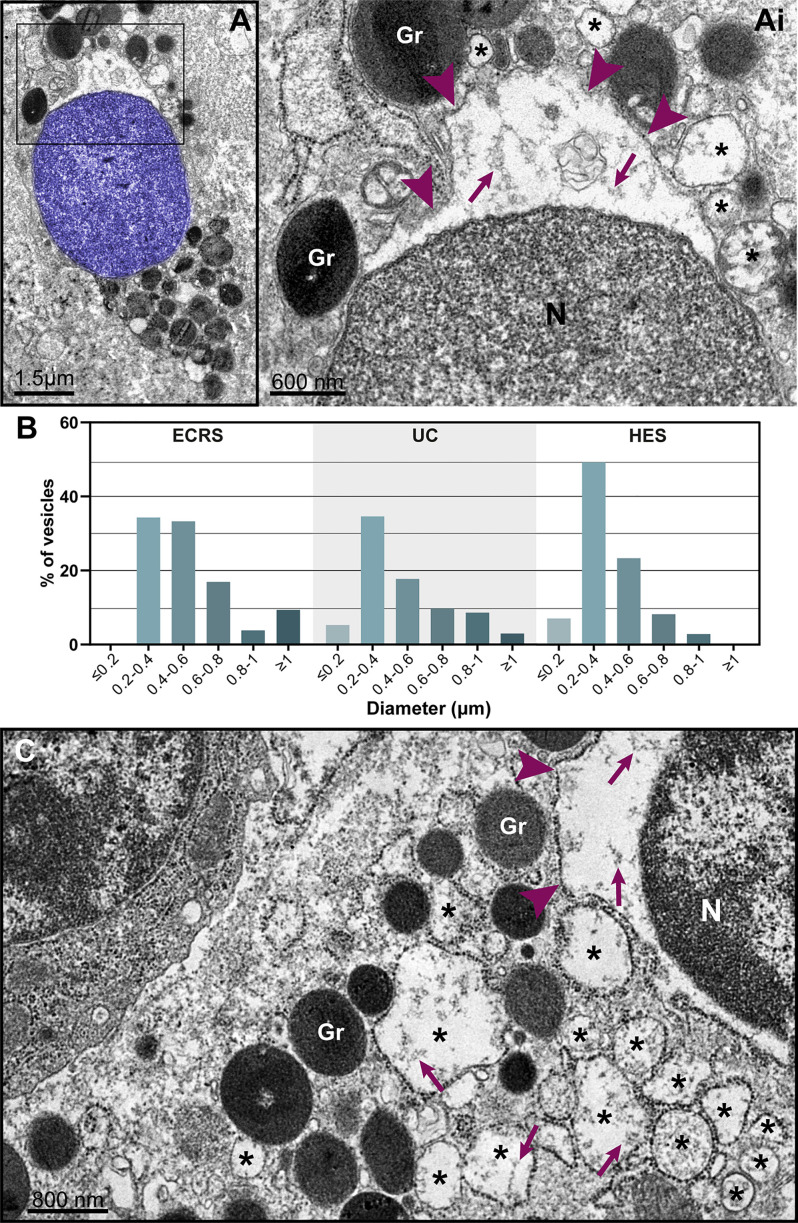
Nuclear envelope alterations associated with ETosis. **(A and Ai)** Typical ETotic eosinophils with decondensed, delobulated, and round nucleus (blue) showing separation of the inner and outer nuclear membranes with enlargement of the perinuclear compartment (boxed area seen in higher magnification in Ai). Arrowheads indicate the external nuclear membrane. Note that the same content (strings, arrows) within the perinuclear space is seen in the lumen of vesicles (*) derived from the nuclear envelope. **(B)** Size range (diameter) of nuclear envelope-originated vesicles in eosinophils undergoing ETosis during EADs. A total of 296 vesicles from ETotic eosinophils were analyzed [92 for eosinophilic chronic rhinosinusitis (ECRS), 88 for ulcerative colitis (UC), and 86 for hypereosinophilic syndrome (HES)]. In **(C)**, a cytolytic non-ETotic eosinophil (nucleus with euchromatin/heterochromatin distinction) showing the same structural changes of the nuclear envelope as seen in **(A)**. Representative images are from biopsy samples (nasal sinuses) from ECRS patients (n = 2) prepared for TEM.

The nuclear envelope also underwent structural changes during *in vivo* EETosis. Tissue-infiltrated eosinophils with round, completely decondensed nuclei showed clear enlargement of the nuclear space, thus leading the two membranes that compose this envelope to become apart (**Figures 5A**, **6A, Ai**) and to form vesicles (**Figure 6Ai**, asterisks) with sizes (diameters) ranging from ~0.2 to 1.0 μm (**Figure 6B**). Nuclear envelope vesiculation was a frequent finding associated with EETosis and vesicles contained the same content aspect as seen within the nuclear space (**Figure 6Ai**). However, we also found changes in the nuclear envelope (separation of the internal and external nuclear membranes and vesiculation) in nuclei from cytolytic eosinophils with no evidence of ETosis, that is, showing nuclei with euchromatin/heterochromatin distinction (**Figure 6C**) thus indicating that these nuclear envelope alterations are not restricted to ETosis.

Interestingly, in contrast to *in vitro* observations reporting that the nuclear envelope breakdown usually precedes plasma membrane rupture (7, 18), our qualitative and quantitative findings showed that the vast majority (100% in ECRS and HES; and 76% in UC) of eosinophils with early signs of ETosis (n = 53 cells) were displaying varied degrees of plasma membrane disruption (ranging from slight loss of integrity to complete rupture or even disappearance). In addition, some nuclei showing typical ETosis features (DDR) but still keeping the nuclear envelope intact were also seen free in the extracellular matrix (**Supplementary Figure S4**). Hence, during *in vivo* ETosis, the process of plasma membrane disruption occurs, in general, in parallel to that of the nuclear envelope.

### Late Signs of EETosis Captured *In Vivo* by TEM

**Figure 7:**
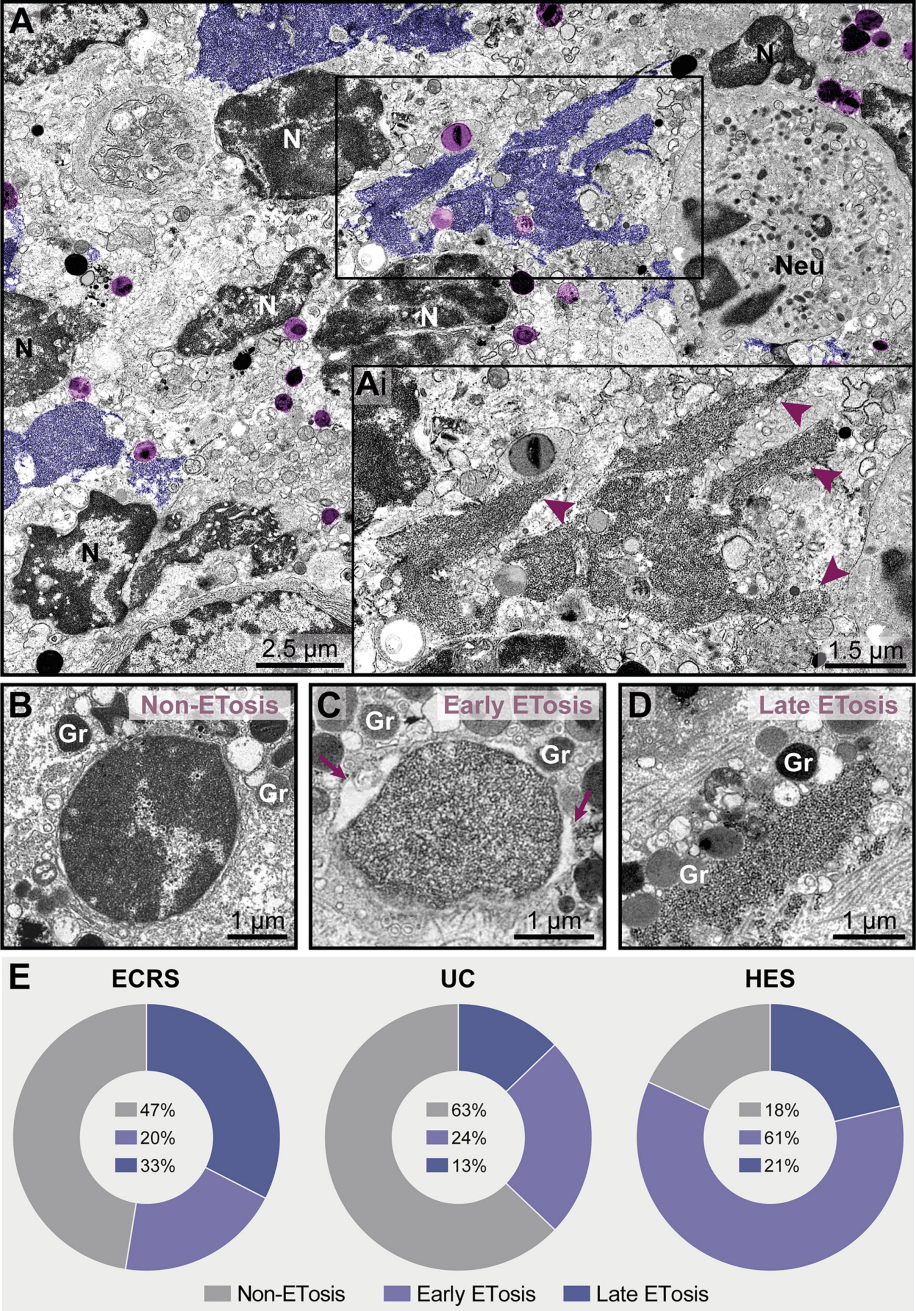
ETosis represents a significant proportion of cytolytic eosinophils in eosinophil-associated diseases (EADs). **(A, Ai)** A tissue inflammatory site [intestinal ulcerative colitis (UC) biopsy] showing ETotic eosinophils with late signs of ETosis represented by decondensed chromatin spread extracellularly as ETs (colored in purple and indicated by arrowheads in Ai at higher magnification). FEGs (light purple) are in contact with ETs. Note that other non-ETotic eosinophils with nuclei (N) exhibiting heterochromatin (condensed)/euchromatin (decondensed) and a neutrophil (Neu) are seen in the same area. **(B–D)** Representative electron micrographs from an ECRS biopsy (nasal sinus tissue) highlighting the structural aspect of nuclei in cytolytic eosinophils. While the presence of condensed/decondensed chromatin denotes a non-ETotic eosinophil, eosinophils in early **(C)** and late **(D)** processes of ETosis show extensively decondensed chromatin. When in early ETosis **(C)**, the nuclear envelope is observed and frequently displays structural changes (enlargement of the perinuclear space, arrows), in late ETosis **(D)**, the nuclear envelope disappears and the chromatin spreads in the extracellular matrix. Note secretory granules (Gr) immersed in the released chromatin. **(E)** Quantitative TEM (total tissue area of 66,000 μm^2^ evaluated), reveals that ~37%-82% of all cytolytic eosinophils in EADs are in process of ETosis and that the proportion of eosinophils in early and late ETosis varies depending on the disease. A total of 151 cytolytic eosinophils with visible nuclear contents [40 for eosinophilic chronic rhinosinusitis (ECRS), 78 for ulcerative colitis (UC), and 33 for hypereosinophilic syndrome (HES)] were evaluated from biopsy samples collected from patients with ECRS (n = 2), UC (n = 16), and HES (n = 2) and prepared for TEM.

**Figure 8:**
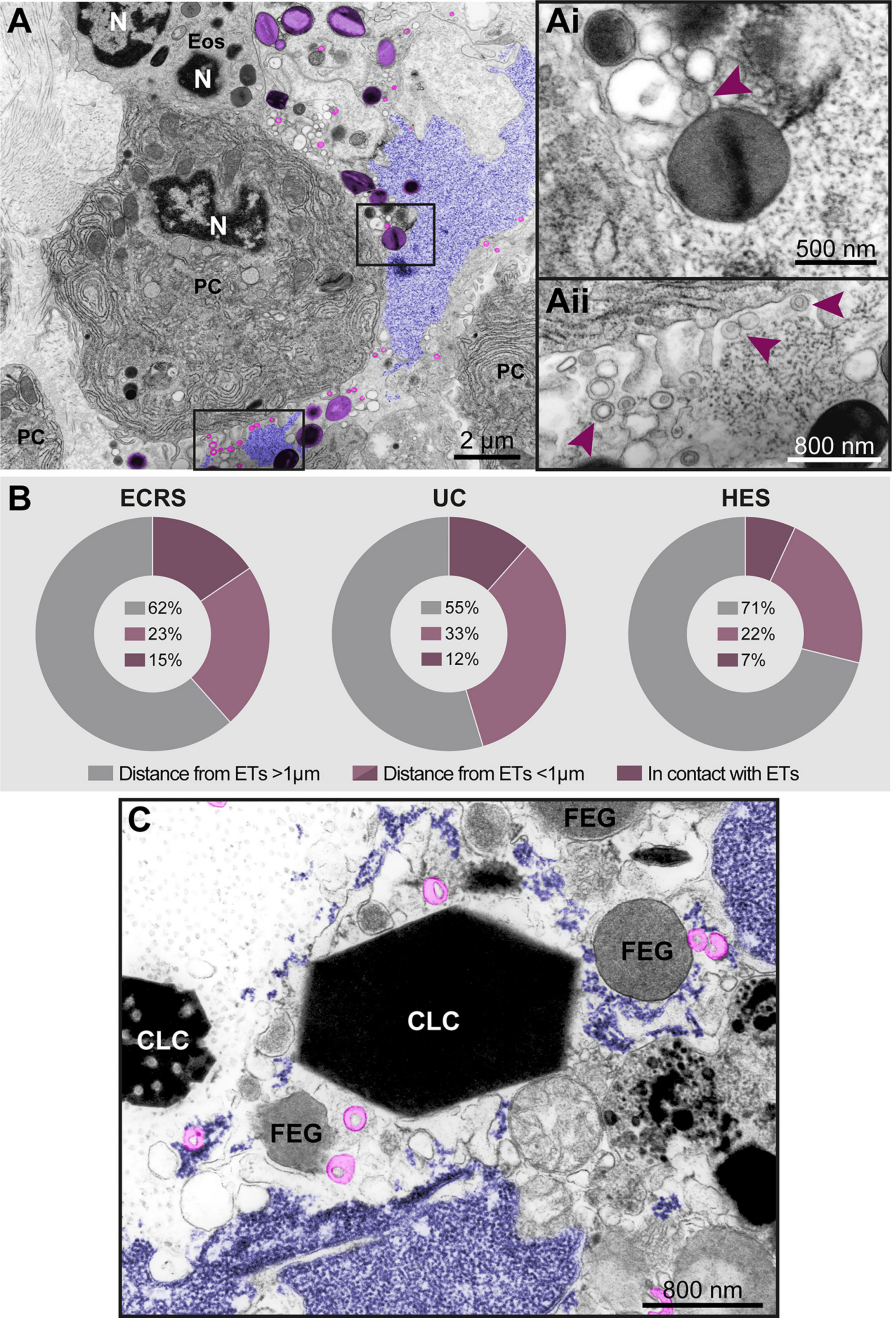
Free-extracellular granules (FEGs), free tubular carriers [eosinophil sombrero vesicles (EoSVs)], and Charcot-Leyden crystals (CLCs) are associated with late ETosis **(A)** An inflamed tissue site (nasal sinus) from a patient with eosinophilic chronic rhinosinusitis (ECRS) shows an ETotic eosinophil with decondensed chromatin (colored in purple) spreading in the extracellular matrix. Note intact secretory granules (light purple) and intact free EoSVs (pink) spread around and in contact with extruded chromatin. The boxed areas in **(A)** are shown in higher magnification in **(Ai, Aii)**. Arrowheads point to typical EoSVs. In **(Ai)**, an EoSVs associates with an intact granule. **(B)** Quantitative TEM of inflammatory tissue sites from all eosinophil-associated diseases (EADs) showed that intact EoSVs are found in direct contact with or close to (<1µm of distance) extruded chromatin, or spread (>1µm of distance from DNA strings) in the tissue area. A total of 744 free EoSVs [335 for ECRS, 181 for ulcerative colitis (UC), and 228 for hypereosinophilic syndrome (HES)] were evaluated in electron micrographs considering only late EETosis. **(C)** CLCs, free EoSVs (pink) and FEGs are enmeshed in tissue chromatin deposits (purple) in a tissue biopsy (nasal sinus) from an ECRS patient. Samples were prepared for TEM.

As noted *in vitro* (**Figure 1G**), the rupture of the nuclear envelope results in chromatin dispersion into the cytoplasm and subsequent release into the extracellular matrix after breakdown of the plasma membrane. Thus, decondensed chromatin emerges from dying eosinophils being released as fibers of DNA and histone complex (EETs) (**Figures 7A, Ai**, see also **Supplementary Figure S5**), which can cover large extracellular areas. Because chromatin has a particular appearance when seen in thin sections, extruded decondensed chromatin was identified in all EADs (**Figures 7** and **8**)

While chromatin spreading as EETs was clearly detected in inflammatory sites, not all cytolytic eosinophils in the same microenvironment exhibited ultrastructural changes of ETosis, thus indicating that different populations of eosinophils might be selectively activated into this pathway. Quantitative analyses scoring all cytolytic eosinophils (ranging from cells with partially to totally disrupted plasma membrane) showing nuclear content (**Figures 7B–D**, n = total of 151 eosinophils) revealed that 53% (ECRS), 37% (UC), and 82% (HES) of them had early (**Figure 7C**) or late (**Figure 7D**) ultrastructural characteristics of ETosis while 47% (ECRS), 63% (UC), and 18% (HES) did not exhibited such features (**Figure 7E**). Moreover, the proportion of eosinophils in early and late process of ETosis varied depending on the disease with the highest proportion of early ETosis being found in HES (**Figure 7E**).

*In vivo* EETosis led not only to the deposition of intact or almost intact secretory granules into the extracellular matrix (**Figures 7Ai** and **8Ai**), but also to the release of eosinophil sombrero vesicles (EoSVs). Free intact EoSVs were associated with FEGs and extracellular chromatin fibers (**Figures 8A, Ai, Aii**). By counting 744 EoSVs free in the extracellular matrix, we found that 7%-15% of these vesicles were immersed in extruded chromatin (direct contact); 22%-33% were located in the proximity of ETs (less than 1 μm of distance), while most of them were still intact but spread in a distance higher than 1 μm from the released chromatin.

**Figure 9:**
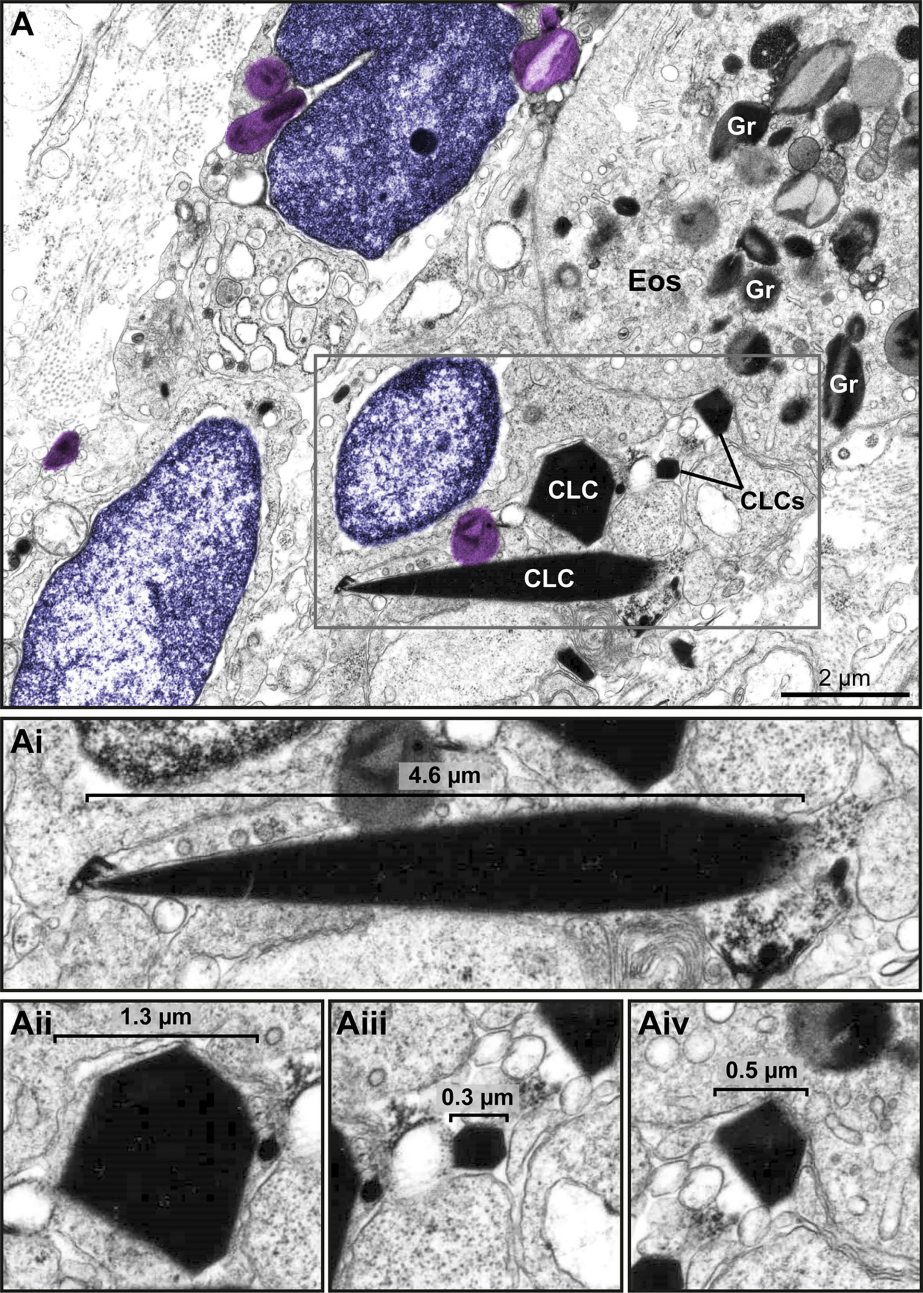
Charcot Leyden crystals (CLCs) are frequently seen in inflammatory sites from eosinophil-associated diseases (EADs) in association with EETosis. **(A)** CLCs displaying varied shapes and sizes are observed by TEM in a biopsy (intestine) of a patient with UC. Large and very small CLCs can be observed in the same microenvironment. Note free-extracellular granules (FEGs, colored in light purple) close to CLCs, an intact eosinophil (Eos), and decondensing nuclei (colored in purple). **(Ai-Aii)** CLCs within the boxed area in **(A)** seen individually in higher magnification to highlight their different morphologies.

Another important finding associated with EETosis was the presence of CLCs, which appeared in inflammatory areas in all EADs. We found typical pyramidal or hexagonal or even amorphous CLCs in the proximity of FEGs (**Supplementary Figure S3** and **Figures 8C** and **9**), externalized chromatin (**Figures 8C**, **9**), and free EoSVs (**Figure 8C**). The notable size variation of CLCs was an interesting observation. By measuring a total of 105 CLCs (24 for ECRS, 61 for UC and 20 for HES), we found a range from 0.3μm to 13.5μm (considering the long axis) and 0.05µm² to 22.4µm² (area), with tiny CLCs being found together with giant CLCs in the same tissue site (**Figure 9**). When the areas of CLCs were compared among the diseases, there was no significant difference among them (mean area of 1.99 ± 0.91, 1.97 ± 0.43, and 1.60 ± 0.39 for ECRS, UC, and HES respectively; *P* > 0.999 (ECRS vs. UC); *P* = 0.998 (ECRS vs. HES); and *P* > 0.99 (UC vs. HES), thus reflecting a consistent formation of CLCs in all eosinophilic diseases.

## Discussion

Cell death manifests with morphological changes. From a morphological point of view, cell death depicts fundamentally a cytolytic or a noncytolytic (apoptotic) morphology and both morphological profiles can be unambiguously distinguished by TEM [reviewed in (6)]. For human eosinophils, cytolysis, as frequently observed in biopsies from patients with EADs, represents much more than a process of cell death. As extensively documented, the presence of cytolytic eosinophils in tissues and secretions, in many circumstances, denotes an important degranulation (secretory) process [reviewed in (22, 23)].

**Figure 10:**
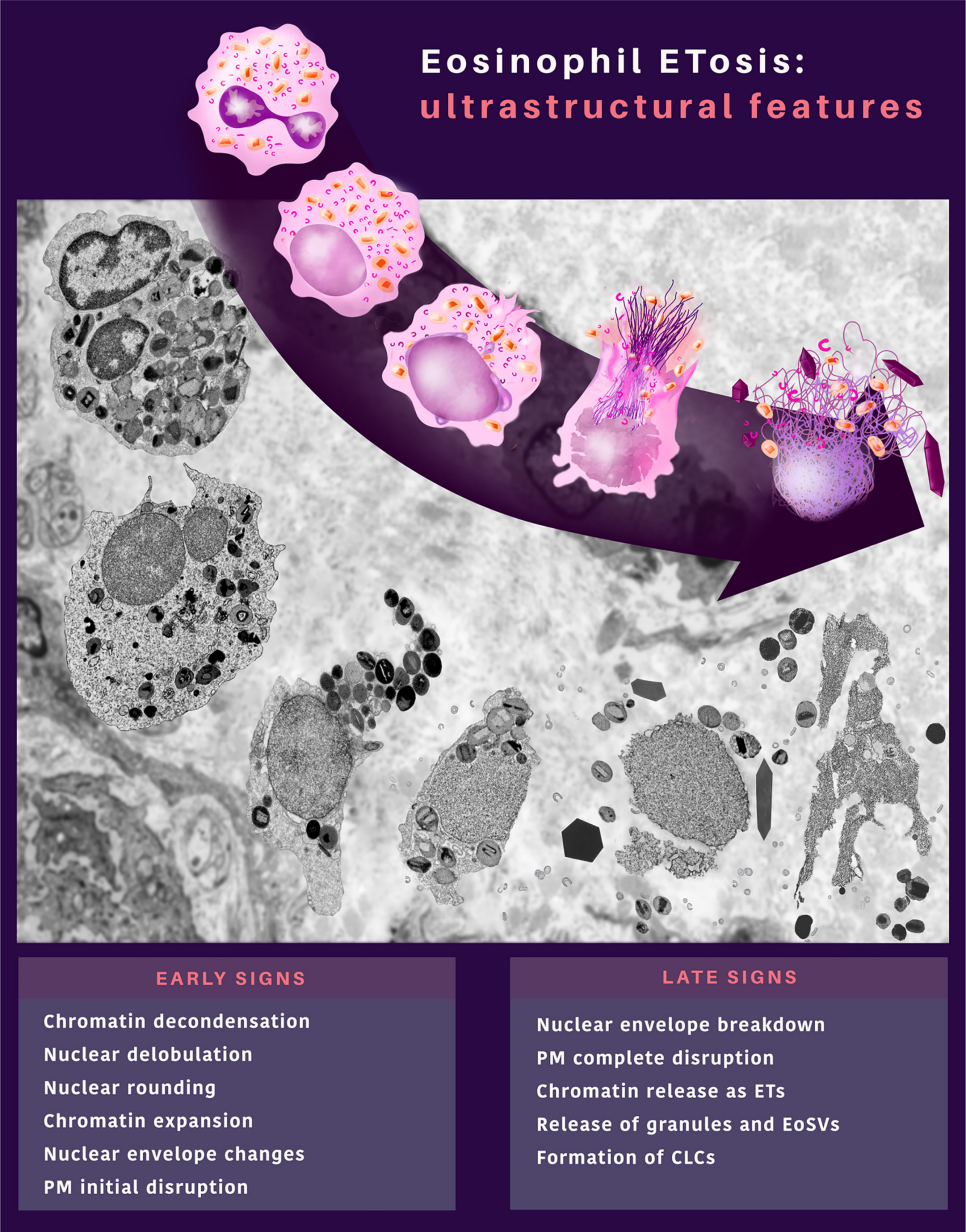
*In vivo* ultrastructural features of eosinophil ETosis (EETosis) observed in tissues during inflammatory human eosinophil-associated diseases. EETosis is characterized by early and late signs, which compose an ultrastructural signature by TEM. Remarkable nuclear changes and initial breakdown of the plasma membrane (PM) indicate early signs of EETosis. The nuclear envelope can show structural changes (vesiculation and enlargement of the perinuclear space between the outer and inner nuclear membrane). Eosinophils at late ETosis show complete disruption of the PM, disruption of the nuclear envelope, and release of secretory granules and EoSVs together with spread chromatin observed as extracellular traps (ETs). Charcot-Leyden crystals (CLCs) are frequently associated with EETosis.

In recent years, eosinophil cytolysis has gained more attention with the recognition of EETosis, a process in which eosinophil lytic death is accompanied by the release of ETs [reviewed in (24)]. Here, we characterized *in vivo* a cytolytic tissue eosinophil profile combined with other ultrastructural hallmarks that can serve as a diagnosis for EETosis at the EM level in biopsies. An ultrastructural EETosis signature (**Figure 10**) was found in three prototypic eosinophilic diseases (ECRS, UC, and HES) in different tissues/organs (nasal sinus, intestine, and skin, respectively), thus, validating the use of TEM as a robust tool to detect EETosis and highlighting this cell process as a common eosinophil secretory mechanism. Although evidence of EETosis has been documented in human tissues (7, 8, 10) and secretions (8, 12, 25) affected by EADs, this is the first time that an EETosis “signature”, that is, a complete description of the ultrastructural aspects of ETosis with early and late signs and quantitative analyses are identified in human tissue biopsies. Moreover, this is the first demonstration of EETosis in an inflammatory bowel disease (ulcerative colitis), indicating that the occurrence of EETosis is a much more common process of eosinophil degranulation.

Based on both qualitative and quantitative analyses, our findings demonstrate that: i) 37-82% of all cytolytic eosinophils in inflammatory sites from different EADs are undergoing ETosis; ii) ETosis manifests with early and late ultrastructural hallmarks but the proportion of eosinophils in early and late process of ETosis varies depending on the disease; iii) ETosis affects isolated or clusters of eosinophils but not all cytolytic eosinophils show ETosis features in the same microenvironment/inflammatory site; iv) EETosis is associated not only with the release of FEGs but also with the extrusion of intact EoSVs and formation of CLCs.

Considering that the release of ETs is the final step in a program of active cytolytic cell death as described for both NETosis (18) and EETosis (7), and that the infiltration of immune cells such as eosinophils in inflammatory tissue sites occurs on a continuum, we sought ultrastructural hallmarks of early ETosis. We found *in vivo* consistent eosinophil morphology compatible with early ETosis, that is, dramatic nuclear changes (DDR) as described *in vitro*. In fact, ETosis is initiated by a particular pattern where the segmented nucleus becomes entirely composed of euchromatin and loses both its euchromatin/heterochromatin distinction and lobular shape. Changes of the nuclear envelope (enlargement of the perinuclear space with separation of the inner and external nuclear membranes and vesiculation) were also, for the first time, detected *in vivo* (**Figure 6**) in association with ETosis. *In vitro*, such ultrastructural alterations were reported for NETosis, after cell activation (18, 26). However, based on our studies, enlargement/vesiculation of the nuclear envelope can also be observed in cytolytic eosinophils with no ETosis signs (**Figure 6C**). Therefore, the disintegration of the nuclear envelope into vesicles can be associated with EETosis but is not an exclusive feature of this cell death process.

One morphological aspect of the ETotic death described *in vitro* is the rupture of the plasma membrane subsequent to that of the nuclear envelope (7, 18). Here, we found that, *in vivo*, the plasma membrane can show different degrees of structural collapse (ranging from a slight loss of integrity to complete rupture or even disappearance) while the nuclear envelope is still intact or starting a process of structural alteration and/or rupture. Because the structural aspects of biological membranes are well observed in morphologic detail only at high visual resolutions (6), our study revealed that, in fact, during *in vivo* ETosis, the plasma membrane disruption initiates, in general, concomitantly with the alteration/breakdown of the nuclear envelope.

Our findings also demonstrate that ETosis can affect part (but not all) of cytolytic eosinophils in inflammatory sites. This suggests that different subpopulations of eosinophils were selectively activated *in vivo* into the ETosis pathway. At baseline conditions, heterogeneity of tissue eosinophils (distinct phenotypic subpopulations) has been demonstrated in the lungs (27) and gastrointestinal tract of both humans and mice (28, 29), thus, indicating that the local microenvironment promotes an eosinophil phenotype associated with tissue-specific functions. Moreover, eosinophil subpopulations have also been identified during diseases, for example, in the skin of patients with varied dermatoses (30). It is also important to consider that the increased trafficking of blood eosinophils into tissues will lead to the presence of eosinophils in different stages/time courses of recruitment and activation. All of these above factors could also explain the variability of ETosis among different eosinophilic diseases. Future studies are needed to better understand how these subpopulations, including infiltrating eosinophils, respond to activation with ET production.

The application of TEM has a further advantage to the study of eosinophil cytolysis/secretory ability. When eosinophil secretory (specific) granules are seen by TEM as FEGs scattered in the extracellular matrix, the identity of their cellular origin, that is eosinophils, is not in question since these granules have a unique internal ultrastructure (5). Thus, the presence of an electron-dense crystalloid, which is found just in eosinophils, identifies eosinophil secretory granules. Here, we found that EETosis *in vivo* is markedly associated with the release of membrane-bound eosinophil granules, which are deposited intact (or almost intact) in the tissues together with EoSVs (**Figures 7**, **8**). Because EoSVs have a typical ultrastructure, they can also be easily identified by TEM (31). It is really intriguing how these vesicles are kept preserved in conjunction with secretory granules in inflammatory sites of all studied EADs, even after the complete disappearance of the plasma membrane and other cellular structures/organelles. Free intact EoSVs were also described in biopsies of patients with other EADs such as eosinophilic esophagitis (EoE) (32). Quantitative ultrastructural analyses from esophageal biopsies of 9 patients with EoE demonstrated that 80% of all eosinophils invading the esophagus (n=900) had evidence of cytolysis and FEGs as well as free EoSVs (32). Cytokines such as IL-4 (33) with its IL-4 alpha chain receptor (34) and interferon-gamma (35) as well as tetraspanins such as CD63, a marker for cell secretion, were localized to EoSVs (36). Moreover, the formation of EoSVs increases significantly in the cytoplasm in activated eosinophils as demonstrated both *in vitro* and *in vivo* [reviewed in (5, 31)]. It is already recognized that FEGs extruded through cytolysis/ETosis can remain functionally active once outside the cell (14, 37). If EoSVs are also active in the extracellular medium it is an interesting aspect of the intriguing eosinophil biology yet to be explored.

Finally, the presence of CLCs in the same tissue site as ETs, FEGs and EoSVs in biopsies of EADs (**Figures 8C**, **9**) drew our attention. CLCs are readily identified by TEM. Our present and previous findings based on ultrastructural analyses of biopsies from EADs in different organs (6) demonstrate that CLCs *in vivo* appear with different morphologies (bipyramidal, hexagonal, or amorphous structures) and with remarkable variation in size, ranging from very large (several micrometers) to very small (lower than 1μm) crystals (**Supplementary Figure S4** and **Figure 9**), which are mostly unnoticeable by light microscopy. CLCs are by products of eosinophilic inflammation and are predominantly made of galectin-10 (Gal-10), a protein that resides as intracellular pools in the peripheral cytoplasm of human eosinophils (38). Our *in vivo* current findings agree with a previous study demonstrating that EETosis mediates CLC formation (14). Substantial amounts of Gal-10 are released under active eosinophil cytolysis, and the increased extracellular concentration of Gal-10 is capable of forming CLCs *in vitro* (14). *In vivo*, CLCs are commonly found in body fluids, secretions, and tissues from patients with EADs, and both CLCs and levels/expression of Gal-10 are considered biomarkers of eosinophil involvement in inflammation (10, 39–41).

In conclusion, our findings identified an ultrastructural signature of EETosis (summarized in **Figure 10**) in prototypic EADs, highlighting the importance of this event as a form of eosinophil degranulation during inflammatory responses. EETosis mediates *in vivo* not only the release of secretory granules but also the extrusion of tubular vesicles (EoSVs) and formation of CLCs, all structures related to eosinophil activation and considered inflammatory markers. Our work helps to understand the processes of eosinophil cell death and deposition of their granules/structures in human EADs contributing to a better characterization of these diseases, fundamental to driving treatments.

## Data Availability Statement

The raw data supporting the conclusions of this article will be made available by the authors, without undue reservation.

## Ethics Statement

The studies involving human participants were reviewed and approved by Committee on Clinical Investigation from Beth Israel Deaconess Medical Center/BIDMC (Boston, MA, USA). The patients/participants provided their written informed consent to participate in this study.

## Author Contributions

RM provided the study supervision and prepared the final version of the manuscript. RM, PW and SU contributed with resources. VN and SU performed experiments. VN, CP, and KB performed imaging analyses. VN prepared the final figures and KB prepared the illustration. All authors contributed in part to writing and editing the manuscript and approved the final version.

## Funding

This work was supported by Conselho Nacional de Desenvolvimento Científico e Tecnológico (CNPq, Brazil, grants 434914/2018-5 and 309734/2018-5 to RM) and Fundação de Amparo à Pesquisa do Estado de Minas Gerais (FAPEMIG, Brazil). CP is a fellowship holder from Coordenação de Aperfeiçoamento de Pessoal de Nível Superior (CAPES, Brazil) and VN was a fellowship holder from Fundação de Amparo à Pesquisa do Estado de Minas Gerais (FAPEMIG, Brazil). This study was also funded in part by a Research Grant on Allergic Disease and Immunology from the Japan Agency for Medical Research and Development (21eK0410055 to SU), a Health Labour Sciences Research Grant (21FE1001 to SU), and JSPS KAKENHI (22K08595, 21K07833, 21K08434, 20H03832, and 20K08794 to SU). Studies were also supported by U.S. National Institutes of Health grants (R01AI020241, R37AI020241) to PW.

## Conflict of Interest

The authors declare that the research was conducted in the absence of any commercial or financial relationships that could be construed as a potential conflict of interest.

## Publisher’s Note

All claims expressed in this article are solely those of the authors and do not necessarily represent those of their affiliated organizations, or those of the publisher, the editors and the reviewers. Any product that may be evaluated in this article, or claim that may be made by its manufacturer, is not guaranteed or endorsed by the publisher.

## References

[1] Valent P Klion AD Horny HP Roufosse F Gotlib J Weller PF. Contemporary Consensus Proposal on Criteria and Classification of Eosinophilic Disorders and Related Syndromes. J Allergy Clin Immunol (2012) 130(3):607–12 e9. doi: 10.1016/j.jaci.2012.02.019 22460074 PMC4091810

[2] O'Sullivan JA Bochner BS. Eosinophils and Eosinophil-Associated Diseases: An Update. J Allergy Clin Immunol (2018) 141(2):505–17. doi: 10.1016/j.jaci.2017.09.022 PMC5803328 29045815

[3] Khoury P Akuthota P Ackerman SJ Arron JR Bochner BS Collins MH. Revisiting the Nih Taskforce on the Research Needs of Eosinophil-Associated Diseases (Re-Tread). J Leukoc Biol (2018) 104(1):69–83. doi: 10.1002/JLB.5MR0118-028R 29672914 PMC6171343

[4] Melo RCN Dvorak AM Weller PF. Contributions of Electron Microscopy to Understand Secretion of Immune Mediators by Human Eosinophils. Microsc Microanal (2010) 16(6):653–60. doi: 10.1017/S1431927610093864 PMC3420811 20875166

[5] Melo RCN Weller PF. Contemporary Understanding of the Secretory Granules in Human Eosinophils. J Leukoc Biol (2018) 104(1):85–93. doi: 10.1002/JLB.3MR1217-476R 29749658 PMC6013358

[6] Melo RCN Dvorak AM Weller PF. Eosinophil Ultrastructure: Atlas of Eosinophil Cell Biology and Pathology. Elsevier (2022). p. 518. doi: 10.1016/C2016-0-04569-2

[7] Ueki S Melo RCN Ghiran I Spencer LA Dvorak AM Weller PF. Eosinophil Extracellular DNA Trap Cell Death Mediates Lytic Release of Free Secretion-Competent Eosinophil Granules in Humans. Blood (2013) 121(11):2074–83. doi: 10.1182/blood-2012-05-432088 PMC3596967 23303825

[8] Ueki S Konno Y Takeda M Moritoki Y Hirokawa M Matsuwaki Y. Eosinophil Extracellular Trap Cell Death-Derived DNA Traps: Their Presence in Secretions and Functional Attributes. J Allergy Clin Immunol (2016) 137(1):258–67. doi: 10.1016/j.jaci.2015.04.041 PMC4674385 26070883

[9] Takeda M Ueki S Yamamoto Y Nara M Fukuchi M Nakayama KI. Hypereosinophilic Syndrome With Abundant Charcot-Leyden Crystals in Spleen and Lymph Nodes. Asia Pacific Allergy (2020) 10(3):e24. doi: 10.5415/apallergy.2020.10.e24 32789109 PMC7402945

[10] Fukuchi M Kamide Y Ueki S Miyabe Y Konno Y Oka N. Eosinophil Etosis-Mediated Release of Galectin-10 in Eosinophilic Granulomatosis With Polyangiitis. Arthritis Reumatol (2021) 73:1683–1693. doi: 10.1002/art.41727 PMC8403105 33750029

[11] Ohta N Ueki S Konno Y Hirokawa M Kubota T Tomioka-Matsutani S. Etosis-Derived DNA Trap Production in Middle Ear Effusion Is a Common Feature of Eosinophilic Otitis Media. Allergol Int (2018) 67(3):414–6. doi: 10.1016/j.alit.2017.11.007 29242145

[12] Echevarria LU Leimgruber C Garcia Gonzalez J Nevado A Alvarez R Garcia LN. Evidence of Eosinophil Extracellular Trap Cell Death in Copd: Does It Represent the Trigger That Switches on the Disease? Int J Chron Obstruct Pulmon Dis (2017) 12:885–96. doi: 10.2147/COPD.S115969 PMC5359000 28352169

[13] Melo RCN Spencer LA Perez SA Neves JS Bafford SP Morgan ES. Vesicle-Mediated Secretion of Human Eosinophil Granule-Derived Major Basic Protein. Lab Invest (2009) 89(7):769–81. doi: 10.1038/labinvest.2009.40 PMC2702460 19398958

[14] Ueki S Tokunaga T Melo RCN Saito H Honda K Fukuchi M. Charcot-Leyden Crystal Formation Is Closely Associated With Eosinophil Extracellular Trap Cell Death. Blood (2018) 132(20):2183–7. doi: 10.1182/blood-2018-04-842260 PMC6238188 30154112

[15] Dvorak A Monahan-Earley R. Diagnostic Ultrastructural Pathology I. In: A Text-Atlas of Case Studies Illustrating the Correlative Clinical-Ultrastructural Pathologic Approach to Diagnosis. Boca Raton: CRC (1992). p. 495 p.

[16] Spencer LA Bonjour K Melo RCN Weller PF. Eosinophil Secretion of Granule-Derived Cytokines. Front Immunol (2014) 5:496. doi: 10.3389/fimmu.2014.00496 25386174 PMC4209865

[17] Melo RCN Weller PF. Piecemeal Degranulation in Human Eosinophils: A Distinct Secretion Mechanism Underlying Inflammatory Responses. Histol Histopathol (2010) 25(10):1341–54. 10.14670/hh-25.1341 PMC3427618 20712018

[18] Fuchs TA Abed U Goosmann C Hurwitz R Schulze I Wahn V. Novel Cell Death Program Leads to Neutrophil Extracellular Traps. J Cell Biol (2007) 176(2):231–41. doi: 10.1083/jcb.200606027 PMC2063942 17210947

[19] Fukuchi M Miyabe Y Furutani C Saga T Moritoki Y Yamada T. How to Detect Eosinophil Etosis (Eetosis) and Extracellular Traps. Allergol Int (2021) 70(1):19–29. doi: 10.1016/j.alit.2020.10.002 33189567 PMC9333458

[20] Barroso MV Gropillo I Detoni M Thompson-Souza GA Muniz VS Figueiredo RT. Structural and Signaling Events Driving *Aspergillus Fumigatus-*Induced Human Eosinophil Extracellular Trap Release. Front Microbiol (2021) 12:633696. doi: 10.3389/fmicb.2021.633696 33679663 PMC7930393

[21] Neubert E Meyer D Rocca F Günay G Kwaczala-Tessmann A Grandke J. Chromatin Swelling Drives Neutrophil Extracellular Trap Release. Nat Commun (2018) 9(1):1–13. doi: 10.1038/s41467-018-06263-5 30218080 PMC6138659

[22] Persson C Uller L. Primary Lysis of Eosinophils as a Major Mode of Activation of Eosinophils in Human Diseased Tissues. Nat Rev Immunol (2013) 13(12):902. doi: 10.1038/nri3341-c1 24270781

[23] Weller PF Spencer LA. Functions of Tissue-Resident Eosinophils. Nat Rev Immunol (2017) 17(12):746–60. doi: 10.1038/nri.2017.95 PMC5783317 28891557

[24] Mukherjee M Lacy P Ueki S. Eosinophil Extracellular Traps and Inflammatory Pathologies-Untangling the Web! Front Immunol (2018) 9:2763. doi: 10.3389/fimmu.2018.02763 30534130 PMC6275237

[25] Omokawa A Ueki S Kikuchi Y Takeda M Asano M Sato K. Mucus Plugging in Allergic Bronchopulmonary Aspergillosis: Implication of the Eosinophil DNA Traps. Allergol Int (2018) 67(2):280–2. doi: 10.1016/j.alit.2017.08.002 28886913

[26] Amulic B Knackstedt SL Abu Abed U Deigendesch N Harbort CJ Caffrey BE. Cell-Cycle Proteins Control Production of Neutrophil Extracellular Traps. Dev Cell (2017) 43(4):449–62, e5. doi: 10.1016/j.devcel.2017.10.013 29103955

[27] Mesnil C Raulier S Paulissen G Xiao X Birrell MA Pirottin D. Lung-Resident Eosinophils Represent a Distinct Regulatory Eosinophil Subset. J Clin Invest (2016) 126(9):3279–95. doi: 10.1172/JCI85664 PMC5004964 27548519

[28] Straumann A Kristl J Conus S Vassina E Spichtin HP Beglinger C. Cytokine Expression in Healthy and Inflamed Mucosa: Probing the Role of Eosinophils in the Digestive Tract. Inflammation Bowel Dis (2005) 11(8):720–6. doi: 10.1097/01.MIB.0000172557.39767.53 16043986

[29] Masterson JC Menard-Katcher C Larsen LD Furuta GT Spencer LA. Heterogeneity of Intestinal Tissue Eosinophils: Potential Considerations for Next-Generation Eosinophil-Targeting Strategies. Cells (2021) 10(2):426. doi: 10.3390/cells10020426 33671475 PMC7922004

[30] Roth N Städler S Lemann M Hösli S Simon HU Simon D. Distinct Eosinophil Cytokine Expression Patterns in Skin Diseases–the Possible Existence of Functionally Different Eosinophil Subpopulations. Allergy (2011) 66(11):1477–86. doi: 10.1111/j.1398-9995.2011.02694.x 21884530

[31] Melo RCN Spencer LA Dvorak AM Weller PF. Mechanisms of Eosinophil Secretion: Large Vesiculotubular Carriers Mediate Transport and Release of Granule-Derived Cytokines and Other Proteins. J Leukoc Biol (2008) 83(2):229–36. doi: 10.1189/jlb.0707503 PMC2734949 17875811

[32] Saffari H Hoffman LH Peterson KA Fang JC Leiferman KM Pease LF 3rd. Electron Microscopy Elucidates Eosinophil Degranulation Patterns in Patients With Eosinophilic Esophagitis. J Allergy Clin Immunol (2014) 133(6):1728–34 e1. doi: 10.1016/j.jaci.2013.11.024 24439077

[33] Melo RCN Spencer LA Perez SA Ghiran I Dvorak AM Weller PF. Human Eosinophils Secrete Preformed, Granule-Stored Interleukin-4 Through Distinct Vesicular Compartments. Traffic (2005) 6(11):1047–57. doi: 10.1111/j.1600-0854.2005.00344.x PMC2715427 16190985

[34] Spencer LA Melo RCN Perez SA Bafford SP Dvorak AM Weller PF. Cytokine Receptor-Mediated Trafficking of Preformed Il-4 in Eosinophils Identifies an Innate Immune Mechanism of Cytokine Secretion. Proc Natl Acad Sci USA (2006) 103(9):3333–8. doi: 10.1073/pnas.0508946103 PMC1413889 16492782

[35] Carmo LAS Bonjour K Spencer LA Weller PF Melo RCN. Single-Cell Analyses of Human Eosinophils at High Resolution to Understand Compartmentalization and Vesicular Trafficking of Interferon-Gamma. Front Immunol (2018) 9:1542. doi: 10.3389/fimmu.2018.01542 30038615 PMC6046373

[36] Carmo LAS Bonjour K Ueki S Neves JS Liu L Spencer LA. Cd63 Is Tightly Associated With Intracellular, Secretory Events Chaperoning Piecemeal Degranulation and Compound Exocytosis in Human Eosinophils. J Leukoc Biol (2016) 100(2):391–401. doi: 10.1189/jlb.3A1015-480R 26965633 PMC6608091

[37] Neves JS Perez SA Spencer LA Melo RCN Reynolds L Ghiran I. Eosinophil Granules Function Extracellularly as Receptor-Mediated Secretory Organelles. Proc Natl Acad Sci USA (2008) 105(47):18478–83. doi: 10.1073/pnas.0804547105 PMC2587599 19017810

[38] Melo RCN Wang H Silva TP Imoto Y Fujieda S Fukuchi M. Galectin-10, the Protein That Forms Charcot-Leyden Crystals, Is Not Stored in Granules But Resides in the Peripheral Cytoplasm of Human Eosinophils. J Leukoc Biol (2020) 108(1):139–49. doi: 10.1002/JLB.3AB0220-311R PMC9318780 32108369

[39] De Re V Simula MP Cannizzaro R Pavan A De Zorzi MA Toffoli G. Galectin-10, Eosinophils, and Celiac Disease. Ann N-Y Acad Sci (2009) 1173:357–64. doi: 10.1111/j.1749-6632.2009.04627.x 19758173

[40] Chua JC Douglass JA Gillman A O'Hehir RE Meeusen EN. Galectin-10, a Potential Biomarker of Eosinophilic Airway Inflammation. PloS One (2012) 7(8):e42549. doi: 10.1371/journal.pone.0042549 22880030 PMC3412795

[41] Furuta GT Kagalwalla AF Lee JJ Alumkal P Maybruck BT Fillon S. The Oesophageal String Test: A Novel, Minimally Invasive Method Measures Mucosal Inflammation in Eosinophilic Oesophagitis. Gut (2013) 62(10):1395–405. doi: 10.1136/gutjnl-2012-303171 PMC3786608 22895393

