# Supplementary material for: In Vivo ETosis of Human Eosinophils: The Ultrastructural Signature Captured by TEM in Eosinophilic Diseases
Source: Front Immunol. 2022 Jul 7;13:938691. doi: 10.3389/fimmu.2022.938691 (PMC9301467; doi:10.3389/fimmu.2022.938691)
Supplement: Supplementary file 1 [file Image_1.pdf]

Supplementary Material – Supplementary Fig. S1

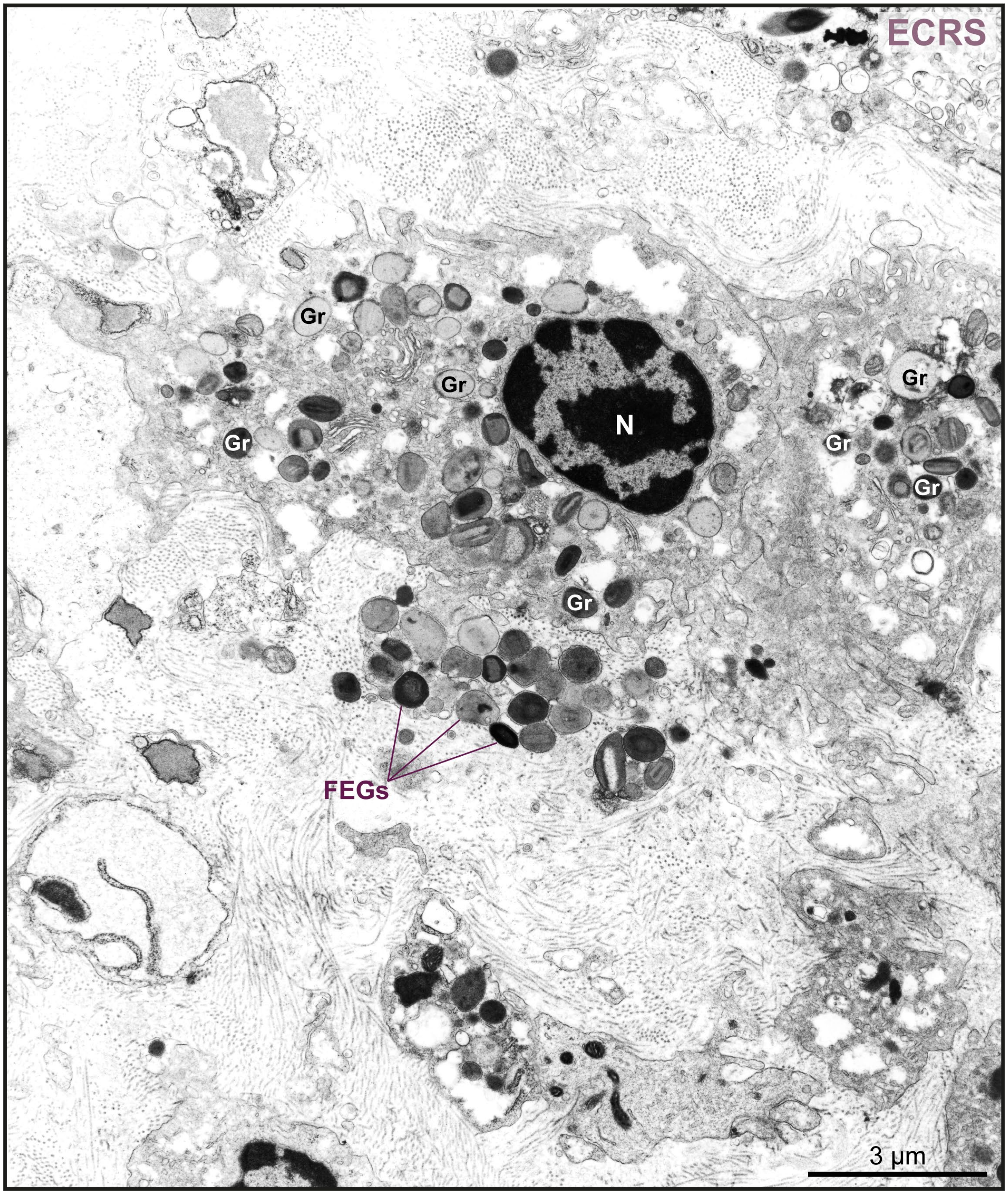

Wide field of Figure 1A, showing the nasal sinus biopsy tissue from a patient with eosinophilic chronic rhinosinusitis (ECRS). N, nucleus; Gr, secretory granules; FEGs, free-extracellular granules.

Supplementary Material – Supplementary Fig. S1

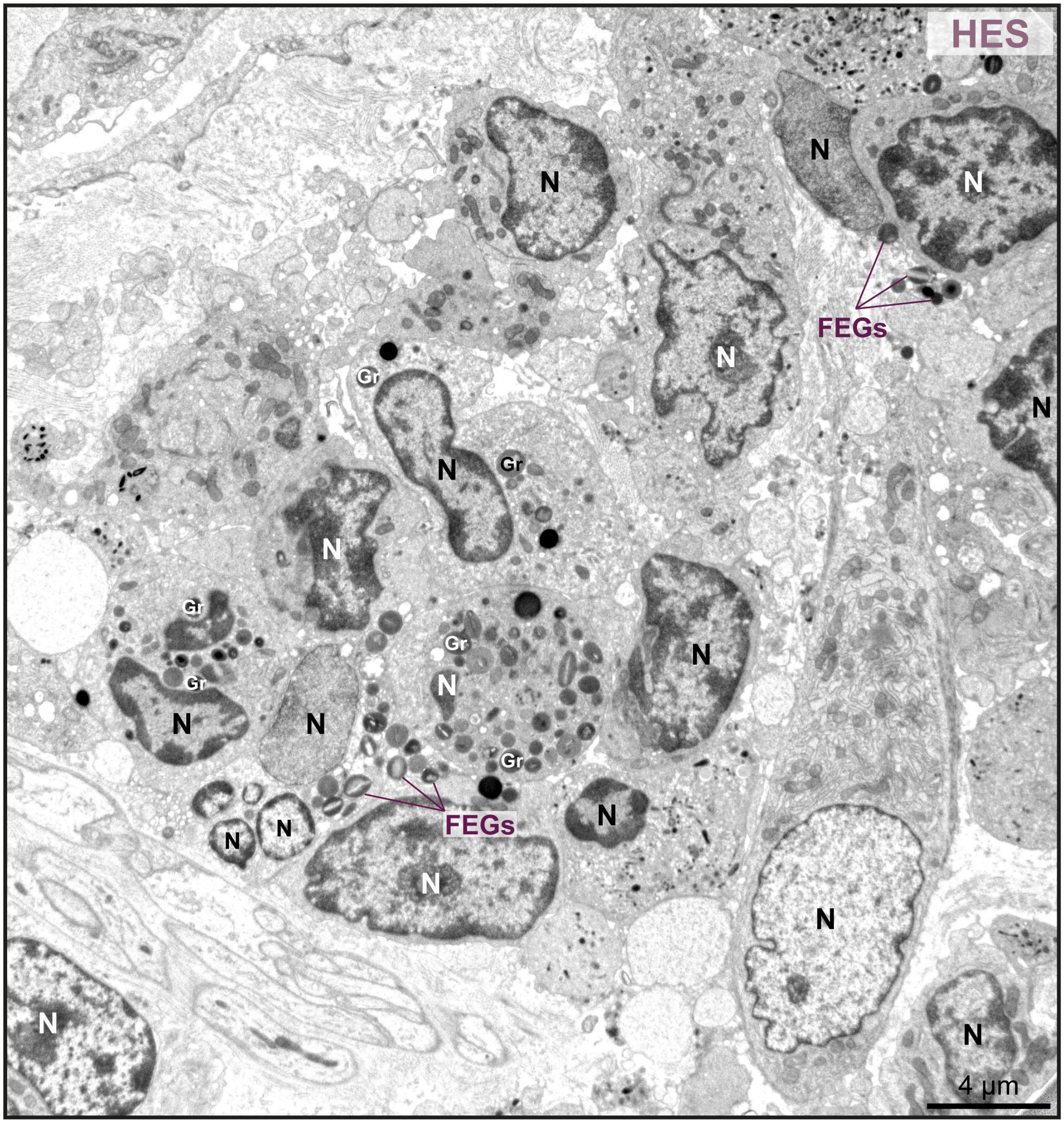

Wide field from Figure 1C, showing the skin biopsy tissue from a patient with hypereosinophilic syndrome (HES). N, nucleus; Gr, secretory granules; FEGS, free-extracellular granules.
