# Supplementary material for: In Vivo ETosis of Human Eosinophils: The Ultrastructural Signature Captured by TEM in Eosinophilic Diseases
Source: Front Immunol. 2022 Jul 7;13:938691. doi: 10.3389/fimmu.2022.938691 (PMC9301467; doi:10.3389/fimmu.2022.938691)
Supplement: Supplementary file 2 [file Image_2.pdf]

Supplementary Material

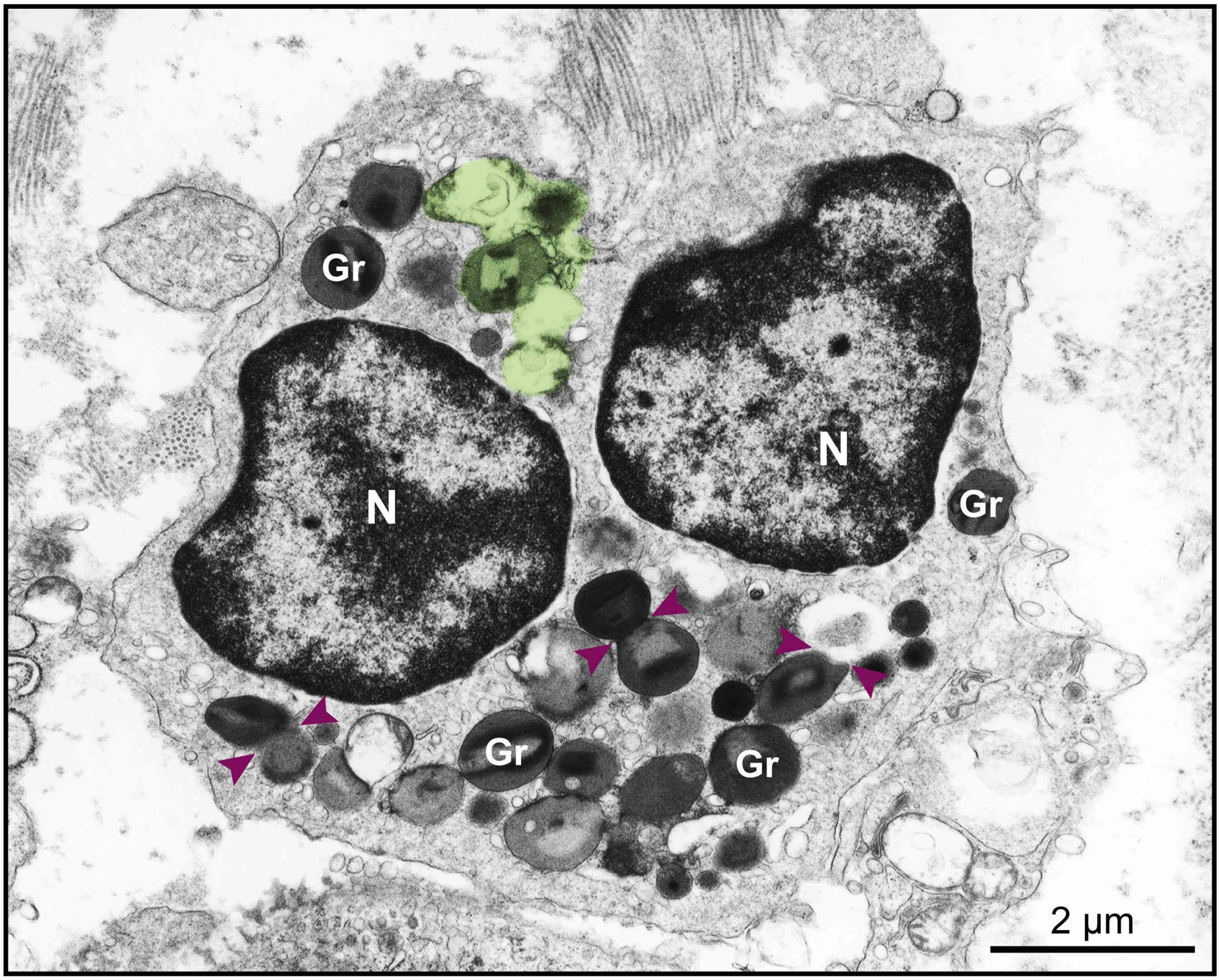

Supplementary Fig. S2. TEM showing a representative mucosal eosinophil in an intestinal biopsy of a o patient with ulcerative colitis. Granule-granule (Gr) fusions (arrowheads) including a large chamber (colored in green) characterize compound exocytosis. Note the bilobed nucleus (N), typical of human eosinophils.
