# Supplementary material for: In Vivo ETosis of Human Eosinophils: The Ultrastructural Signature Captured by TEM in Eosinophilic Diseases
Source: Front Immunol. 2022 Jul 7;13:938691. doi: 10.3389/fimmu.2022.938691 (PMC9301467; doi:10.3389/fimmu.2022.938691)
Supplement: Supplementary file 4 [file Image_4.pdf]

Supplementary Material

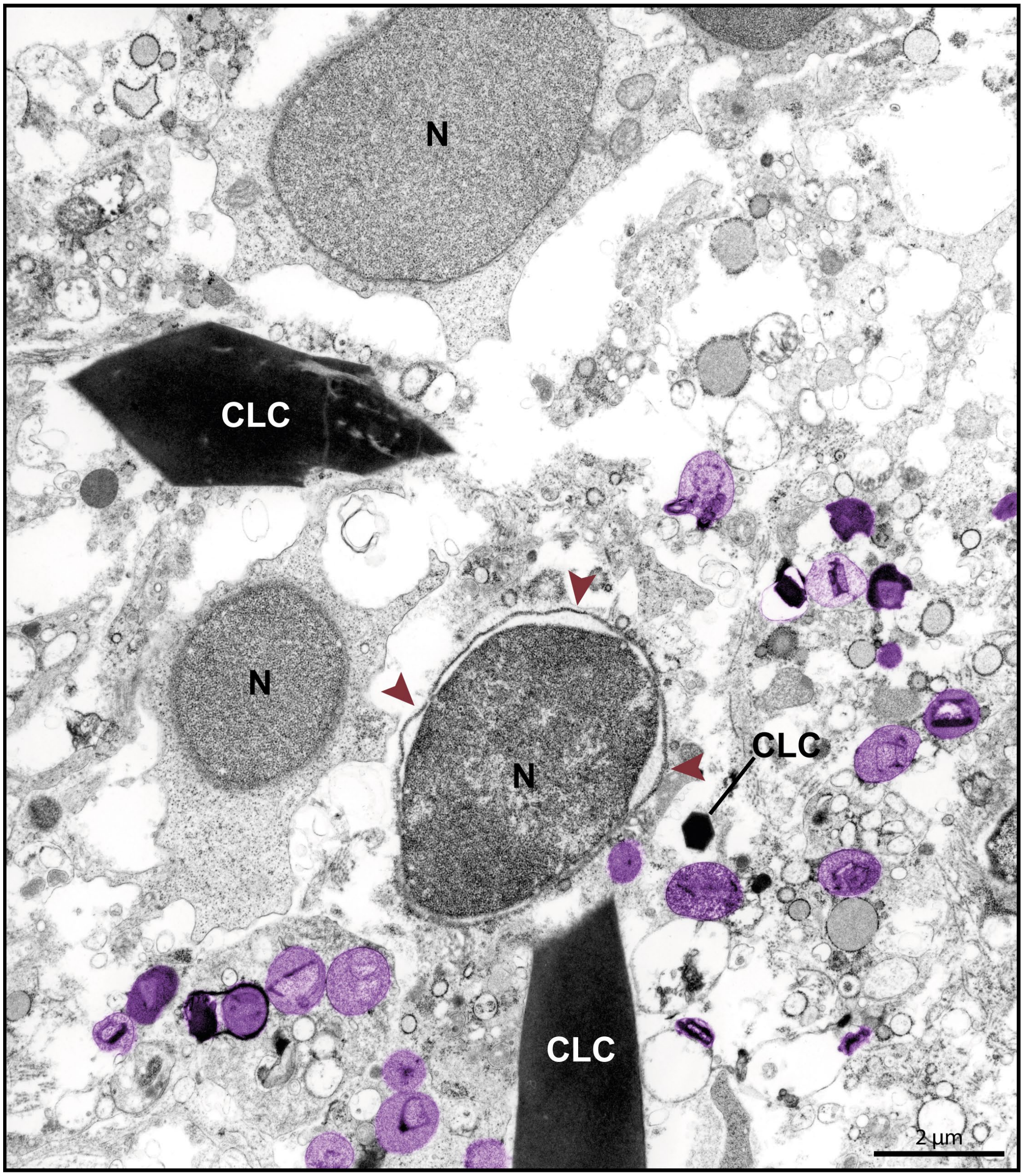

Supplementary Fig. S4. EETosis showing free decondensed, delobulated and rounded nuclei (N) deposited in an inflammatory intestinal biopsy from a patient with ulcerative colitis. Note that nuclear envelope is not disrupted but show structural alterations with enlargement of the nuclear space between the inner and external membranes (arrowheads). The deposition of FEGs (purple) and varied sized CLCs can also be observed.
