# Supplementary material for: In Vivo ETosis of Human Eosinophils: The Ultrastructural Signature Captured by TEM in Eosinophilic Diseases
Source: Front Immunol. 2022 Jul 7;13:938691. doi: 10.3389/fimmu.2022.938691 (PMC9301467; doi:10.3389/fimmu.2022.938691)
Supplement: Supplementary file 5 [file Image_5.pdf]

Supplementary Material – Supplementary Fig. S5

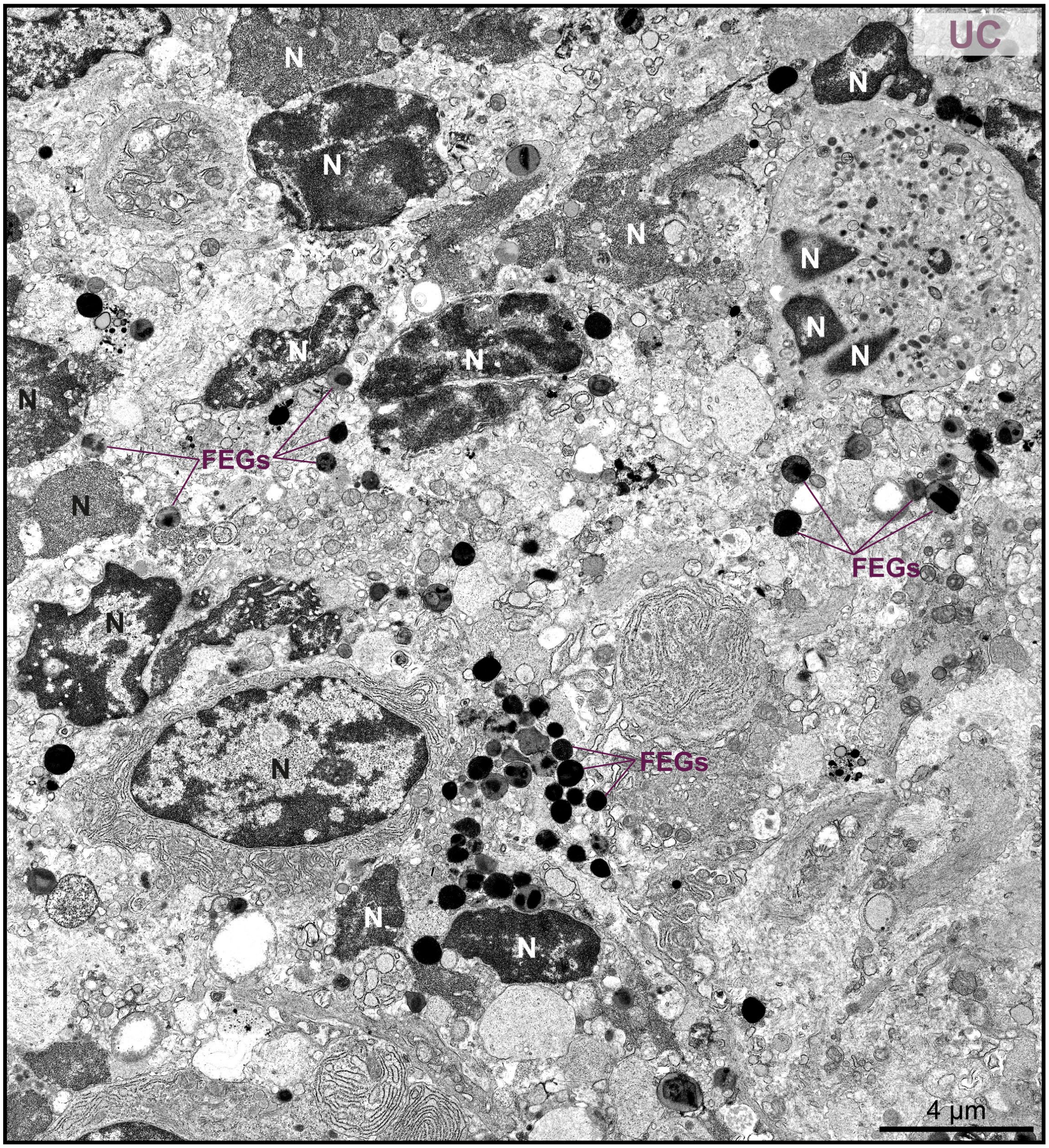

Wide Field of Figure 7A, showing the intestinal tissue biopsy from a patient with ulcerative colitis (UC). N, nucleus; FEGs, free-extracellular granules.
